# Selective, genetically induced increase in synaptic vesicle priming

**DOI:** 10.1126/sciadv.aee6848

**Published:** 2026-04-08

**Authors:** Mohammad Aldahabi, Flora Balint, Andrea Lorincz, Noa Lipstein, Nils Brose, Zoltan Nusser

**Affiliations:** ^1^ Laboratory of Cellular Neurophysiology, HUN-REN Institute of Experimental Medicine, Budapest 1083, Hungary.; ^2^ Leibniz-Forschungsinstitut für Molekulare Pharmakologie (FMP), 13125 Berlin, Germany.; ^3^ Department of Molecular Neurobiology, Max Planck Institute for Multidisciplinary Sciences, Göttingen 37075, Germany.

## Abstract

Synaptic vesicle (SV) release probability (*Pv*) is determined by two probabilistic factors: the probability of release sites being occupied by fusion-competent, well-primed SVs and their fusion probability (*P*_fusion_). While recent studies emphasize SV priming as a key mechanism underlying functional synaptic diversity, disentangling priming from fusion is notoriously challenging. Here we developed a mouse genetic approach for inducible and selective increase of SV priming. A histidine-to-lysine mutation at position 567 of Munc13-1 increases its function. Combining this mutation with a Cre-dependent removal of the wild-type Munc13-1 allele enables cell type–selective enhancement of Munc13-1 function. This manipulation increased excitatory postsynaptic current amplitude at hippocampal synapses exclusively through elevating *Pv* without affecting release site number or quantal size. A sequential, two-step priming model predicts that the enhanced *Pv* results from an elevated proportion of well-primed SVs, without altering *P*_fusion_. Last, we provide unequivocal evidence that the postsynaptic target cell type–dependent variability in presynaptic glutamate release is mainly the consequence of variability in SV priming.

## Teaser

Selective increase of synaptic vesicle priming reveals the mechanisms of functional synaptic diversity.

## INTRODUCTION

Action potential (AP)–triggered synaptic vesicle (SV) exocytosis is a stochastic process, reflecting the probabilistic nature of the molecular reactions that control SV docking, priming, and fusion at the presynaptic active zone (AZ). At the same time, these processes are tightly regulated, resulting in cell type– and synaptic input–specific differences in SV release probabilities (*Pv*) and short-term plasticity (STP) patterns. Theoretical and experimental studies demonstrated that functional synaptic diversity increases the robustness and computational power of neuronal networks (*1*, *2*). Resolving the mechanisms underlying functional synaptic diversity is crucial not only for understanding neuronal network function in health and disease but also essential for developing targeted molecular manipulations to determine causal relationships between SV priming/fusion and neuronal network functions.

Variability in the amplitude of postsynaptic responses could arise from variations in the number of release sites (*N*), quantal size (*q*), and *Pv*. During complex, temporally structured presynaptic activities, altering *N* or *q* results in a linear scaling of the postsynaptic response, while changing *Pv* alters the dynamics of release (i.e., STP). Moreover, *Pv* exhibits a hundred-fold variability across synapses, indicating that *Pv* is the key parameter determining synaptic diversity (*3*). The traditional view of SV release primarily considers those processes that lead to the exocytosis of fusion-ready SVs docked at the AZ. However, theoretical and experimental studies over the past decades highlighted a dynamic and reversible SV priming process (*4*, *5*). The reversibility of priming introduces an additional layer of probability: the probability that an SV release site is occupied by a fusion-competent, well-primed SV (*P*_occupancy_). Consequently, *Pv* is now considered as the product of two independent probabilistic processes: *P*_occupancy_ and the probability that such well-primed SVs will fuse with the plasma membrane upon the arrival of an AP (*P*_fusion_) (*6*). Recent studies demonstrated that variability in the proportion of well-primed SVs underlies variations in *Pv* in many central synapses (*7*–*9*) and dynamic changes in *P*_occupancy_ during repetitive presynaptic activities, rather than in *P*_fusion_, cause distinct STP patterns (*5*, *7*, *10*–*19*). Moreover, the induction of long-term potentiation at cortical and hippocampal excitatory synapses can be accounted for by an increase in the proportion of well-primed SVs without a change in *P*_fusion_ (*20*–*22*). Dissecting the contributions of priming and fusion to SV release is therefore more than just a conceptual challenge; uncovering the molecular mechanisms that differentiate these processes is essential for enabling their selective manipulation and for establishing causal links between SV priming/fusion and neuronal network function.

SV fusion critically depends on the density, conductance, and open probability of presynaptic voltage-gated Ca^2+^ channels [VGCCs; mainly N- and P/Q type in cortical networks (*23*–*25*)]; the Ca^2+^ sensitivity of the Ca^2+^ sensor of SV release [mainly synaptotagmin-1 (*26*)]; the distance between VGCCs and the release sensor; and the biophysical properties and concentration of Ca^2+^ buffers surrounding the release sites and VGCCs (*3*, *27*–*30*). SV priming, however, requires a distinct set of proteins, mainly the so-called soluble *N*-ethylmaleimide–sensitive factor attachment protein receptor (SNARE) complex regulators, which include Munc13, Munc18, CAPSs, RIM and synaptotagmins (*31*–*33*). Among these, Munc13-1 has emerged as an essential SV priming protein; its genetic deletion leads to the total loss of primed SVs and abolishes SV release (*32*–*34*). The activity of Munc13-1 is regulated by multiple proteins and second messengers, including RIM1/2, Ca^2+^/calmodulin, diacylglycerol (DAG), and Ca^2+^/phosphatidylinositol 4,5-bisphosphate (*35*–*37*). The functional significance of corresponding regulatory domains of Munc13-1 in synaptic transmission and plasticity has been extensively studied (*35*, *36*, *38*–*40*). It has been postulated that the activation of its C1 domain, either with DAG or DAG analogs such as phorbol 12,13-dibutyrate (PDBU), facilitates the full assembly of SNARE complex and increases SV release (*41*). Mutation of amino acid residue 567 of Munc13-1 from histidine (H) to lysine (K; termed here as HK) mimics the disinhibition of Munc13-1 by DAG binding to the C1 domain (*42*) and can be considered as a gain-of-function mutation (*37*, *43*).

The aim of the present study was to exploit this mutation on SV priming and fusion (*44*). To reveal the functional effect of HK mutation, we created triple mutant mice by crossing three mouse lines: (i) mice carrying a heterozygous HK mutation of Munc13-1 [Munc13-1^(HK/+)^ (*43*)]; (ii) mice with a floxed Munc13-1 allele enabling Cre-dependent deletion [Munc13-1^(fl/fl)^ (*44*)], and (iii) mice heterozygous for Munc13-2 deletion [Munc13-2^(+/−)^; Cre-independent (*45*)]. Crossing Munc13-1^(HK/+)^ with Munc13-1^(fl/fl)^ mice was required because homozygous Munc13-1^(HK/HK)^ offspring are nonviable (*43*). By injecting Cre recombinase–encoding adeno-associated virus (AAVs) into the dorsal hippocampus of Munc13-1^(HK/fl)^ or Munc13-1^(+/fl)^ mice [on either Munc13-2^(+/+)^ or Munc13-2^(−/−)^ background], we achieved temporally controlled and selective manipulation of Munc13-1 function in synapses containing either wild-type or HK mutant Munc13-1, each expressed from a single allele. We verified the functional consequences of this point mutation on hippocampal CA1 pyramidal cell (PC)–fast spiking interneuron (FSIN) synapses with paired whole-cell recordings, quantal analysis, and model fitting. We also applied super resolution stimulated emission depletion (STED) microscopy of Munc13-1–immunolabeled hippocampal sections to reveal the effect of this point mutation on Munc13-1 nanoclusters within presynaptic AZs. Last, by comparing the effects of this mutation at CA1PC-FSIN and CA1PC–oriens-lacunosum-moleculare (O-LM) interneuron (IN) synapses, we provide unequivocal evidence that the postsynaptic target cell type–dependent variability in presynaptic *Pv* and STP is mainly the consequence of large difference in the probability that a release site is occupied by a fusion-competent SV (*P*_occupancy_) rather than *P*_fusion_.

## RESULTS

### Munc13-1 HK mutation increases evoked EPSC amplitudes at CA1PC-FSIN connections

**Fig. 1.**
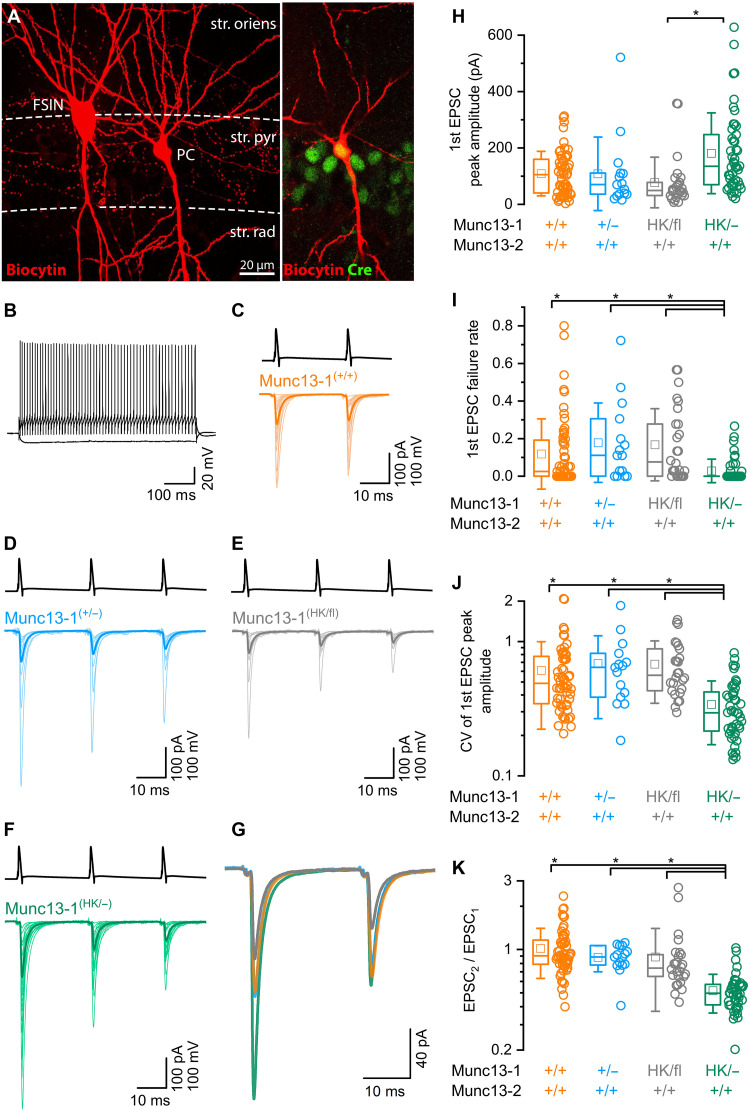
Munc13-1 HK point mutation increases the amplitudes of evoked EPSCs at CA1PC-FSIN connections. (**A**) Confocal maximum intensity projection image of a biocytin-filled, synaptically connected PC-FSIN pair in the hippocampal CA1 region (left). Cre-recombinase is expressed in the biocytin-filled PC (right). str. oriens, stratum oriens; str. pyr, stratum pyramidale; str. rad, stratum radiatum. (**B**) Membrane potential responses of the IN to depolarizing and hyperpolarizing current pulses showing fast spiking firing characteristics. (**C** to **F**) Evoked AP responses at 40 Hz in CA1 PCs (black trace) expressing either homozygous wild-type Munc13-1^(+/+)^ (C), one allele of Munc13-1^(+/−)^ (D), heterozygous Munc13-1^(HK/fl)^ (E), or only the point-mutated Munc13-1^(HK/−)^ (F). Corresponding evoked EPSCs are shown in FSINs (orange, blue, gray, and green, respectively). Averaged EPSC traces are shown from individual pairs (light traces), and superimposed is their grand total average (GTA, dark traces; *n* = 56, 15, 26, and 46 pairs, respectively). All genotypes share Munc13-2^(+/+)^ background. (**G**) Superimposed GTA traces from Munc13-1^(+/+)^ (orange), Munc13-1^(+/−)^ (blue), Munc13-1^(HK/fl)^ (gray), and Munc13-1^(HK/−)^ (green) CA1PCs-FSINs. (**H**) The peak amplitudes of the first eEPSCs are significantly larger in Munc13-1^(HK/−)^ (green, *n* = 46) compared to Munc13-1^(HK/fl)^ (gray, *n* = 26, *P* = 0.00019). (**I**) Failure rates are significantly lower in Munc13-1^(HK/−)^ compared to Munc13-1^(+/+)^, Munc13-1^(+/−)^, and Munc13-1^(HK/fl)^ genotypes (*P* = 0.022, 0.004, and 0.0003, respectively). (**J**) CV values of the first eEPSC amplitudes are significantly smaller in Munc13-1^(HK/−)^ compared to Munc13-1^(+/+)^, Munc13-1^(+/−)^, and Munc13-1^(HK/fl)^ genotypes (*P* = <0.0001, 0.00076, and <0.0001, respectively). (**K**) Paired-pulse ratios are significantly smaller in Munc13-1^(HK/−)^ compared to Munc13-1^(+/+)^, Munc13-1^(+/−)^, and Munc13-1^(HK/fl)^ genotypes (*P* = <0.0001, <0.0001, and 0.00012, respectively). Kruskal-Wallis statistical test (KW test) with Dunn’s post hoc test was used in (H) to (K). *P < 0.05.

For selective and inducible genetic manipulation of Munc13-1 function, we used Munc13-1^(HK/fl)^ mice on a Munc13-2^(+/+)^ background. Adult mice received Cre recombinase– and mCherry-expressing AAVs injections into the CA1 region of the dorsal hippocampus. This induced the deletion of the floxed wild-type Munc13-1 allele, leaving only the point-mutated Munc13-1 allele intact. We refer to these Cre-expressing CA1PCs as Munc13-1^(HK/−)^ PCs. To test the effects of the HK mutation on postsynaptic responses, simultaneous whole-cell paired recordings were performed in acute hippocampal slices at least 3 weeks after virus injection, from mCherry-positive presynaptic CA1PCs and postsynaptic FSINs. Recordings were conducted in the presence of a low-affinity competitive glutamate receptor antagonist [0.35 mM γ-D-Glutamylglycine (γ-DGG)] to prevent postsynaptic receptor saturation. Evoked excitatory postsynaptic currents (eEPSCs) were recorded from FSINs in response to APs generated in Munc13-1^(HK/−)^ PC (Fig. 1, A, B, and F) and compared to PCs of other Munc13-1 genotypes: wild-type [Munc13-1^(+/+)^; Fig. 1C], heterozygous Munc13-1^(+/−)^ [(after Cre-mediated deletion of one floxed allele in Munc13-1^(+/fl)^ animals; Fig. 1D], and Munc13-1^(HK/fl)^ (in the absence of Cre-mediated deletion; Fig. 1E). All these Munc13-1 genotypes were on a Munc13-2^(+/+)^ background. The mean eEPSC amplitudes were significantly larger in Munc13-1^(HK/−)^ (181.2 ± 143.4 pA, *n* = 46) compared to Munc13-1^(HK/fl)^ (mean: 77.5 ± 89.7 pA, *n* = 26) condition (Fig. 1H). The larger initial eEPSCs in Munc13-1^(HK/−)^ synapses were accompanied by a decrease in failure rates (Fig. 1I), coefficients of variation (CV; Fig. 1J), and paired pulse ratios (Fig. 1K) consistent with a presynaptic locus of change. Following paired recordings, the slices were fixed in an aldehyde-containing fixative and the intracellular biocytin and Cre-recombinase were visualized. Throughout the study, only post hoc verified Cre-recombinase immunopositive presynaptic PCs were included in the Munc13-1^(HK/−)^ and Munc13-1^(+/−)^ data (Fig. 1A).

A previous study from our laboratory has shown that Munc13-2 is preferentially localized to presynaptic AZs synapsing onto mGluR1α-expressing dendrites (e.g., O-LM INs) but is absent from AZs targeting PV-expressing dendrites [e.g., FSINs; (*46*)]. The absence of Munc13-2 in AZs innervating FSINs predicted the lack of functional effects of removing a Munc13-2 allele. We tested this by performing paired recordings between PC and FSIN from Munc13-1^(+/−)^ and Munc13-1^(HK/−)^ mice on Munc13-2^(+/+)^ and Munc13-2^(+/−)^ background. Initial eEPSC peak amplitude, CV, and failure rate did not differ significantly between Munc13-1^(+/−)^/Munc13-2^(+/+)^ and Munc13-1^(+/−)^/Munc13-2^(+/−)^ mice (fig. S1, A to C). We also performed the same comparison for Munc13-1^(HK/−)^/Munc13-2^(+/+)^ and Munc13-1^(HK/−)^/Munc13-2^(+/−)^ mice and again observed no significant differences in these three measured parameters (fig. S1, D to F). These results demonstrate that removing one Munc13-2 allele does not affect CA1PC-FSIN synaptic transmission. Therefore, both Munc13-2^(+/+)^ and Munc13-2^(+/−)^ animals were used in subsequent electrophysiological experiments involving Munc13-1^(HK/−)^ CA1PC-FSIN synapses.

### Munc13-1 HK mutation increases *Pv* at CA1PC-FSIN connections, but not *N* or *q*

**Fig. 2.**
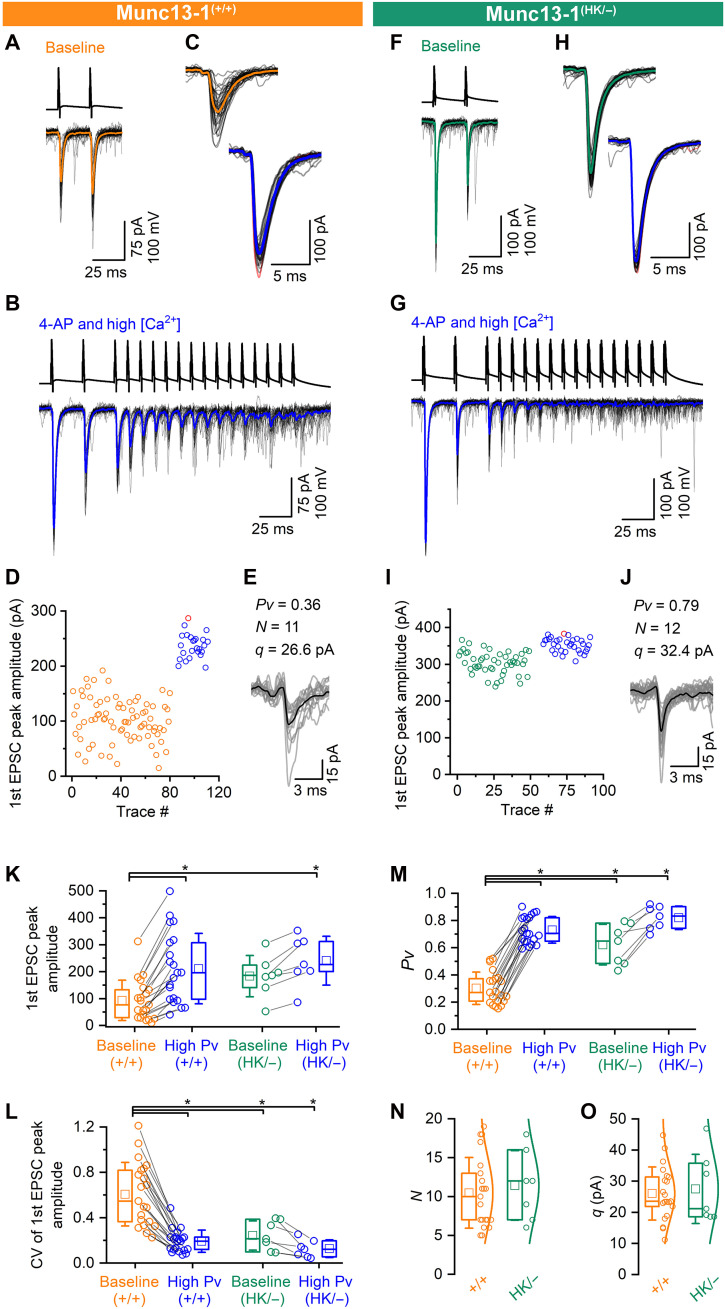
Munc13-1 HK mutation increases *Pv* without altering *N* or *q*. (**A**) eEPSCs recorded under baseline condition from an FSIN (individuals, gray; average, orange) in response to two presynaptic APs (black) in a wild-type PC. (**B**) After baseline, 5 μM 4-AP and high [Ca^2+^]_e_ were washed in (high-*Pv* period), and stimulation was switched to a train of APs. (**C**) First eEPSCs from baseline (A) and high-*Pv* (B) periods (individual eEPSCs, gray; mean eEPSCs, orange and blue; largest eEPSCs, red). (**D**) Stability plot of first eEPSC amplitudes in baseline (orange) and high-*Pv* (blue) conditions (red symbol, largest eEPSC). (**E**) Individual, potentially uniquantal eEPSCs (gray) recorded at the end of the 100-Hz train were averaged (black) to obtain *q*. *N* was calculated by dividing the largest eEPSC amplitude by the *q*. *Pv* was calculated by dividing the first eEPSC mean amplitude (baseline and high-*Pv* conditions) with the largest eEPSC amplitude. (**F** to **J**) The same as (A) to (E) but recorded from Munc13-1^(HK/−)^ CA1 PCs. (**K**) First eEPSC amplitudes are significantly smaller in Munc13-1^(+/+)^ baseline (orange) compared to the high-*Pv* (blue, *P* = 0.004) and Munc13-1^(HK/−)^ high-*Pv* (blue, *P* = 0.005) conditions. (**L**) CV of the first eEPSC amplitudes is significantly higher in Munc13-1^(+/+)^ baseline (orange) compared to the high-*Pv* (blue, *P* = <0.0001) and Munc13-1^(HK/−)^ baseline (green, *P* = 0.036) and high-*Pv* (blue, *P* = <0.0001) conditions. (**M**) The *Pv* is significantly smaller in Munc13-1^(+/+)^ baseline (orange) compared to high-*Pv* (blue; *P* = <0.0001) and Munc13-1^(HK/−)^ baseline (green, *P* = 0.038) and high-*Pv* (blue, *P* = <0.0001) conditions. (**N** and **O**) The calculated *N* (N) and *q* (O) are not significantly different (*N*: *P* = 0.59; *q*: *P* = 1, MW test) between Munc13-1^(+/+)^ and Munc13^(HK/−)^ conditions. KW test with Dunn’s post hoc test was used unless otherwise stated. *P < 0.05.

**Fig. 3.**
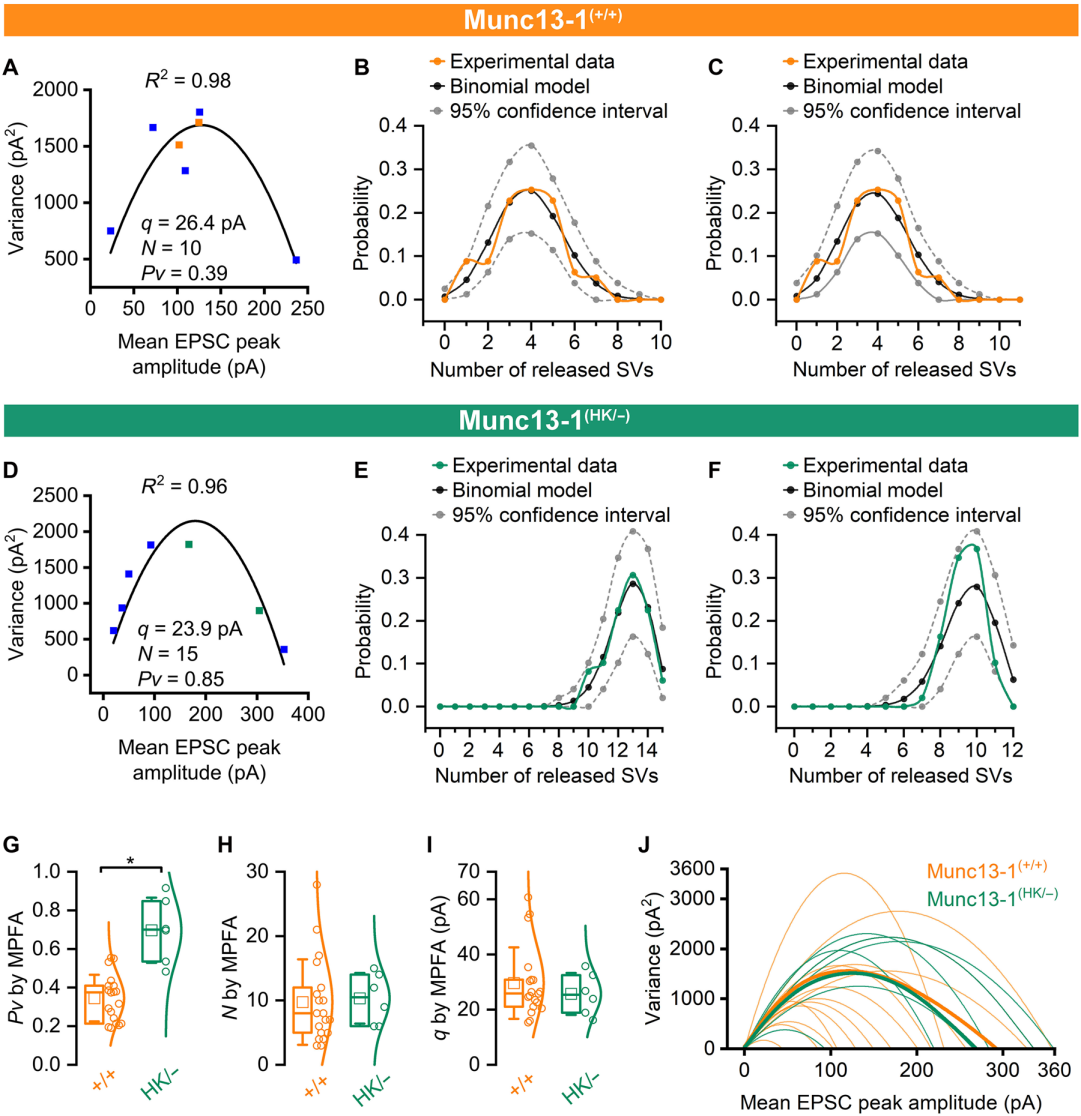
MPFA demonstrates that Munc13-1 HK mutation increases *Pv* at CA1PC-FSIN connections without altering *N* and *q*. (**A**) Relationship between mean and variance values of eEPSC peak amplitudes recorded from Munc13-1^(+/+)^ mice. Data are from the same pair shown in Fig. 2 (A and B). The mean and variance of eEPSC peak amplitudes were calculated for the baseline paired-pulse recordings (orange squares) and for the first four eEPSCs under high-*Pv* conditions (blue squares). *N* and *q* were estimated from the parabolic fit. *Pv* was calculated by dividing the mean of first eEPSC amplitudes in baseline condition by *N* and *q*. (**B** and **C**) The probabilities of the number of released quanta are plotted for the experimental data (orange) and for a binomial distribution calculated with *N* and *Pv* (black) as determined with MPFA (B) or with the method described in Fig. 2 (C). The 95% confidence intervals of the binomial distributions are also shown (gray). The data are within the 95% confidence interval, indicating that the release can be adequately approximated with binomial statistics. (**D** to **F**) The same as (A) to (C) but for the Munc13^(HK/−)^ CA1PC-FSIN pair shown in Fig. 2 (F and G). (**G**) The calculated initial baseline *Pv* using MPFA in Munc13-1^(+/+)^ is significantly lower (*P* = 0.001, MW test) compared to that in Munc13-1^(HK/−)^. (**H** and **I**) The *N* and *q* as calculated with MPFA are not significantly different (*P* = 0.48 and 0.87, respectively, MW test) between Munc13-1^(+/+)^ and Munc13^(HK/−)^ populations. (**J**) Individual parabolic fits (thin lines) to mean versus variance plots are shown for Munc13-1^(+/+)^ (orange) and Munc13-1^(HK/−)^ (green) CA1PC-FSIN pairs. Averages of all parabolic fits [thick lines, *n* = 19 for Munc13-1^(+/+)^, showing only 13 individual fits, and *n* = 6 for Munc13-1^(HK/−)^] are very similar, indicating similar *N* and *q*. *P < 0.05.

To identify the locus of alteration in transmission, we performed quantal analysis of eEPSCs in wild-type and Munc13-1^(HK/−)^ CA1PC-FSIN synapses (Figs. 2 and 3). First, we recorded eEPSCs in response to two presynaptic APs (interstimulus interval: 25 ms) for 6 min in 2 mM extracellular Ca^2+^ concentration ([Ca^2+^]_e_, wild-type baseline, Fig. 2, A, C, and D; Munc13-1^(HK/−)^ baseline, Fig. 2, F, H, and I). Next, we elevated the release by increasing the [Ca^2+^]_e_ to 4 to 8 mM and by washing in the K^+^ channel blocker 4-aminopyridine (4-AP; 5 μM). In addition, we switched to a stimulation protocol consisting of two APs at 40 Hz followed by a long burst of 15 APs at 100 Hz, repeated every 5 s (Fig. 2, B and G). This manipulation increased the first eEPSC amplitude from 93.2 ± 74.9 to 211.3 ± 130.9 pA at wild-type synapses and from 183.6 ± 76.9 to 240.7 ± 90.3 pA at Munc13-1^(HK/−)^ synapses (Fig. 2K), accompanied by a reduction in CV (Fig. 2L). Quantal size was measured at the end of the 100-Hz train when the failure rates were high because of the depletion of the SV pool and the likelihood of multiple quanta released simultaneously was low. The mean amplitude of these presumed uniquantal eEPSCs was considered as the *q* (Fig. 2, E and J, see Materials and Methods). We calculated the *N* by dividing the amplitude of the largest eEPSC (red traces in Fig. 2, C and H and red points in Fig. 2, D and I) by the *q* (see Materials and Methods). *Pv* was calculated at both baseline and high-*Pv* conditions by dividing the mean amplitude of the first eEPSCs by the maximal eEPSC amplitude observed during the high-*Pv* period.

The mean baseline *Pv* for the first eEPSC, calculated for HK mutated Munc13-1 CA1PC-FSIN connections, (0.62 ± 0.15; *n* = 7) was significantly higher compared to that obtained from wild-type Munc13-1 CA1PC-FSIN synapses (0.30 ± 0.12, *n* = 21; Fig. 2M). Application of the potassium channel blocker and high [Ca^2+^]_e_ significantly increased the *Pv* to 0.73 ± 0.10 in wild-type mice. In Munc13-1^(HK/−)^ CA1PC-FSIN connections, our pharmacological manipulation also resulted in a small increase in *Pv* (0.82 ± 0.09), which did not reach significance (Fig. 2M). The calculated *N* and *q* in point-mutated (*N* = 11.4 ± 4.5; *q* = 27.5 ± 11.1 pA) and wild-type connections (*N* = 10.5 ± 4.6, *q* = 26.1 ± 8.5 pA; Fig. 2, N and O) were similar.

Multiple probability fluctuation analysis [MPFA; (*47*)] is a widely used quantal analysis method based on the assumption that the stochastic properties of SV release from presynaptic terminals can be described with binomial statistics. For a binomial distribution, the mean and the variance have a parabolic relationship. Therefore, by imposing distinct *Pv*s at a synapse and fitting a parabola to the plot of mean versus variance of eEPSC peak amplitudes, one can estimate the quantal parameters. Using the quantal estimated parameters (*N* and *Pv*) in Fig. 2 and with MPFA (Fig. 3), we generated binomial distributions and performed bootstrapping to estimate confidence intervals around the means accounting for the variability due to the limited number of trials (see Materials and Methods). These comparisons (Fig. 3, B, C, E, and F) revealed that our experimental data followed binomial distributions remarkably well.

A parabolic fit to the variance versus mean EPSC amplitude plots resulted in similar *N* (HK/−: 10.3 ± 3.9; wild-type: 9.7 ± 6.7; Fig. 3H) and *q* (HK/−: 25.7 ± 7.6 pA; wild-type: 29.6 ± 13.0 pA; Fig. 3I) and a significantly larger initial *Pv* (HK/−: 0.70 ± 0.17; wild-type: 0.35 ± 0.12; Fig. 3G) estimates for the point-mutated CA1PC-FSIN connections. Two independent quantal analysis methods implied that differences in eEPSC amplitudes between wild-type and point-mutated CA1PC-FSIN synapses are the consequence of a selective increase in *Pv*, without any alteration in *N* or *q*.

The Munc13-1^(HK/−)^ synapses had a larger initial eEPSC amplitude but stronger paired-pulse depression (PPD) compared to wild-type synapses (Fig. 1, H and K). To calculate the size of readily releasable pool (RRP) of SVs, we applied a linear back-extrapolation method (*48*) to data obtained from wild-type and Munc13-1^(HK/−)^ synapses (as shown in Fig. 2, B and G, high-*Pv* train). The estimated RRP size was not significantly different between the point-mutated and wild-type CA1PC-FSIN synapses [*P* = 0.3 Mann-Whitney *U* test (MW) test; HK/−: 14.4 ± 7.7 SVs, *n* = 10; wild-type: 17.8 ± 8.3 SVs, *n* = 21].

### A sequential, two-step priming model indicates an increase in the proportion of well-primed SVs in Munc13-1 HK–mutated synapses

**Fig. 4.**
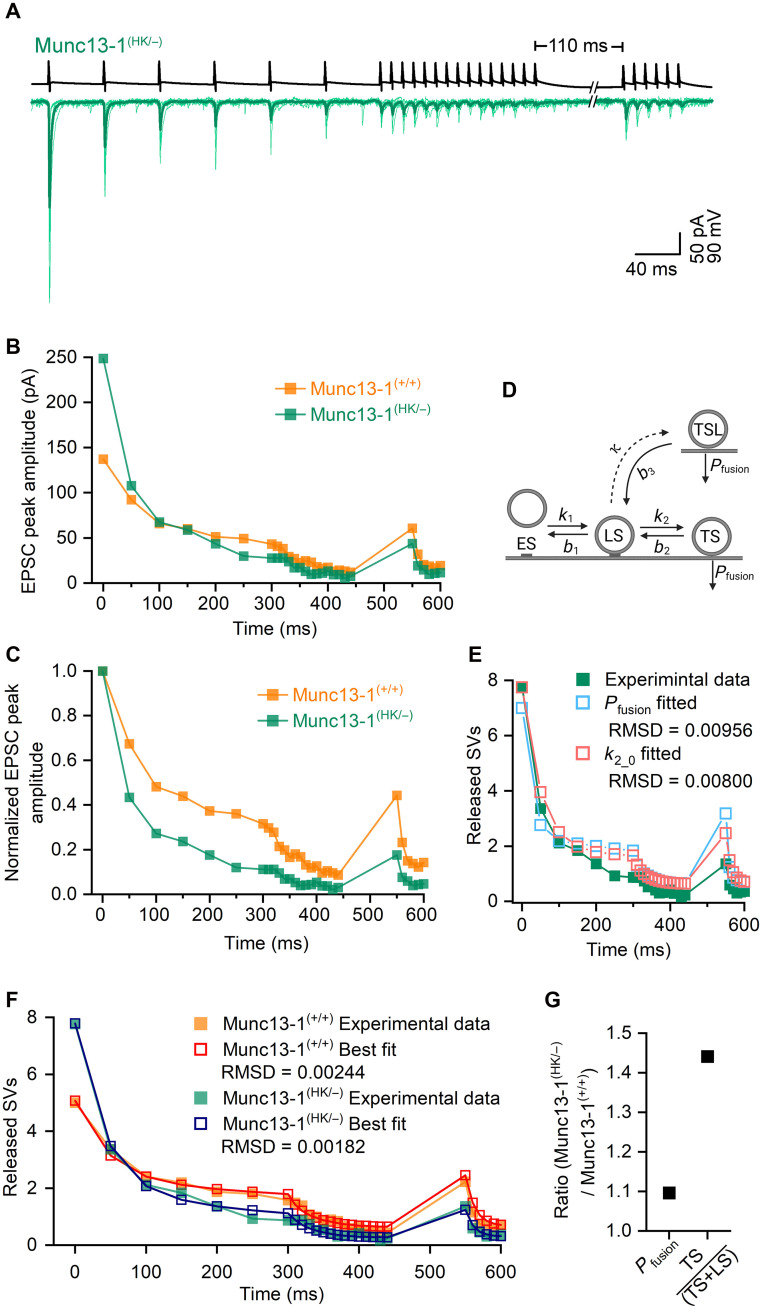
A sequential, two-step SV priming model indicates an increase in fraction of well-primed SVs in Munc13-1^(HK/−)^ CA1PC-FSIN connections. (**A**) A complex train of presynaptic stimulation in Munc13-1^(HK/−)^ PC (black trace) evokes EPSCs in FSIN (green traces). Averaged EPSC traces from individual pairs are shown (light green), and superimposed is the GTA of nine recorded pairs (dark green). (**B**) The peak amplitudes of the GTA eEPSCs versus time are plotted for Munc13-1^(HK/−)^ (green) and Munc13-1^(+/+)^ (orange) CA1PC-FSIN connections. Munc13-1^(+/+)^ data are from (Fig. 1G) (*9*). (**C**) Evoked EPSC amplitudes were normalized to the amplitude of the first EPSC of the train in Munc13-1^(HK/−)^ and Munc13-1^(+/+)^ mice, demonstrating differences in the STP. (**D**) Schematic illustration of the sequential, two-step SV priming model containing empty sites (ES), loosely docked (LS), tightly docked (TS), and labile TS (TSL) SVs. SVs can be released from the TS and TSL states, these states differ in their stability. State transition represented by dashed line indicate instantaneous transitions, while those represented by solid lines occur with rate constants. (**E**) The results of model simulations are shown (open symbols) superimposed to the experimental data (green filled symbols). For the simulation, model parameter values were adopted from the best fit to the wild-type CA1PC-FSIN data [from (*9*)]. Either only *P*_fusion_ (blue) or only *k*_2_0_ (red) was optimized, while all other parameters were kept unchanged. Optimizing *k*_2_0_ resulted in a smaller error (RMSD) compared to that when *P*_fusion_ was fitted. (**F**) Experimental data and the best model fit for Munc13-1^(+/+)^ and Munc13-1^(HK/−)^ CA1PC-FSIN connections are superimposed. (**G**) Ratios of *P*_fusion_ and TS fraction [TS/(TS + LS)] in Munc13-1^(HK/−)^ versus Munc13-1^(+/+)^ demonstrate a 45% larger TS fraction at Munc13-1^(HK/−)^ CA1PC-FSIN connection compared to wild type.

To explore mechanisms through which Munc13-1 HK mutation increases *Pv*, first, we evoked postsynaptic EPSCs in FSINs with a complex train of presynaptic APs, consisting of a 20-Hz preconditioning train, a long high-frequency train (15 APs at 100 Hz), and a short, high-frequency train (6 APs at 100 Hz) following a recovery period of 110 ms (Fig. 4A). When compared to wild-type CA1PC-FSIN connections [data obtained from (*9*)], eEPSCs at Munc13-1^(HK/−)^ synapses exhibited an approximately 65% larger amplitude for the first AP (Fig. 4B) and showed a more robust short-term depression (STD) during both the 20 Hz and the subsequent 100-Hz trains (Fig. 4C). At the end of the 100-Hz train, the responses were dominated by release failures, resulting in very small mean eEPSC amplitudes (9.0 pA; Fig. 4, A to C). After the high-frequency train, the recovery after 110 ms was also slower in the point mutated pairs (18% of the first eEPSC) compared to wild-type connections (44%; Fig. 4C).

A previous study from our laboratory demonstrated that parameters of a sequential, two-step priming model can be reliably estimated by fitting the model to postsynaptic responses evoked by such a complex presynaptic activation protocol (*9*). We started by using the parameter values that best fit the control wild-type data as our initial guesses for the point-mutated data. First, we allowed the optimization of only *P*_fusion_ (Fig. 4, D and E), which resulted in a ~40% increase in this parameter (from 0.6 to 0.83). However, this model fit failed to recapitulate the PPD and vastly overestimated the recovery after 110 ms (Fig. 4E). Next, the rate constant of the second priming step (*k*_2_0_, rate constant from loosely docked (LS) to tightly docked (TS) states: LS → TS transition at rest) was selectively optimized. The model better reproduced STD during the 20- and 100-Hz trains, resulting in a somewhat smaller root mean squared deviation (RMSD), but the recovery was still overestimated (Fig. 4E). Because optimizing *P*_fusion_ or *k*_2_0_ alone could not explain the differences between Munc13-1^(HK/−)^ and wild-type synapses, we fitted the model to the Munc13-1^(HK/−)^ data by allowing all parameters (except the Ca^2+^-related ones and *N*_total_) to vary and compared the resulting parameter set with that obtained from the wild-type synapses (Fig. 4F, parameter values are listed in table S1). Comparing the parameters of the best fits to wild-type and mutant data, we found that *P*_fusion_ is similar (wild-type: 0.6, HK/−: 0.66), but an increase in the proportion of well-primed SVs (TS fraction = TS/(LS + TS); wild-type: 0.44, point HK/−: 0.64) mainly accounts for the enhanced eEPSC amplitudes (Fig. 4G). The results of our modeling suggest that the increase in *Pv* in Munc13-1 point-mutated synapses is primarily the consequence of an increased proportion of well-primed SVs (*P*_occupancy_) rather than increasing the *P*_fusion_.

### The effect of PDBU is occluded by the HK mutation of Munc13-1

We have previously used PDBU for selective pharmacological increase of SV priming (*P*_occupancy_ or TS fraction) and 4-AP to selectively enhance *P*_fusion_ (*8*, *9*). The HK point mutation in the C1 domain prevents DAG/PDBU binding (*37*, *43*). Thus, if PDBU exerts its effect solely through Munc13-1 in hippocampal slices, PDBU-induced potentiation should be abolished at CA1PC-FSIN synapses expressing the DAG/PDBU binding–deficient HK point mutation. To test this, we conducted pharmacological experiments using Munc13-1^(HK/−)^ CA1PC-FSIN pairs with Munc13-1^(+/−)^ as controls, ensuring that both groups have only a single Munc13-1 allele.

**Fig. 5.**
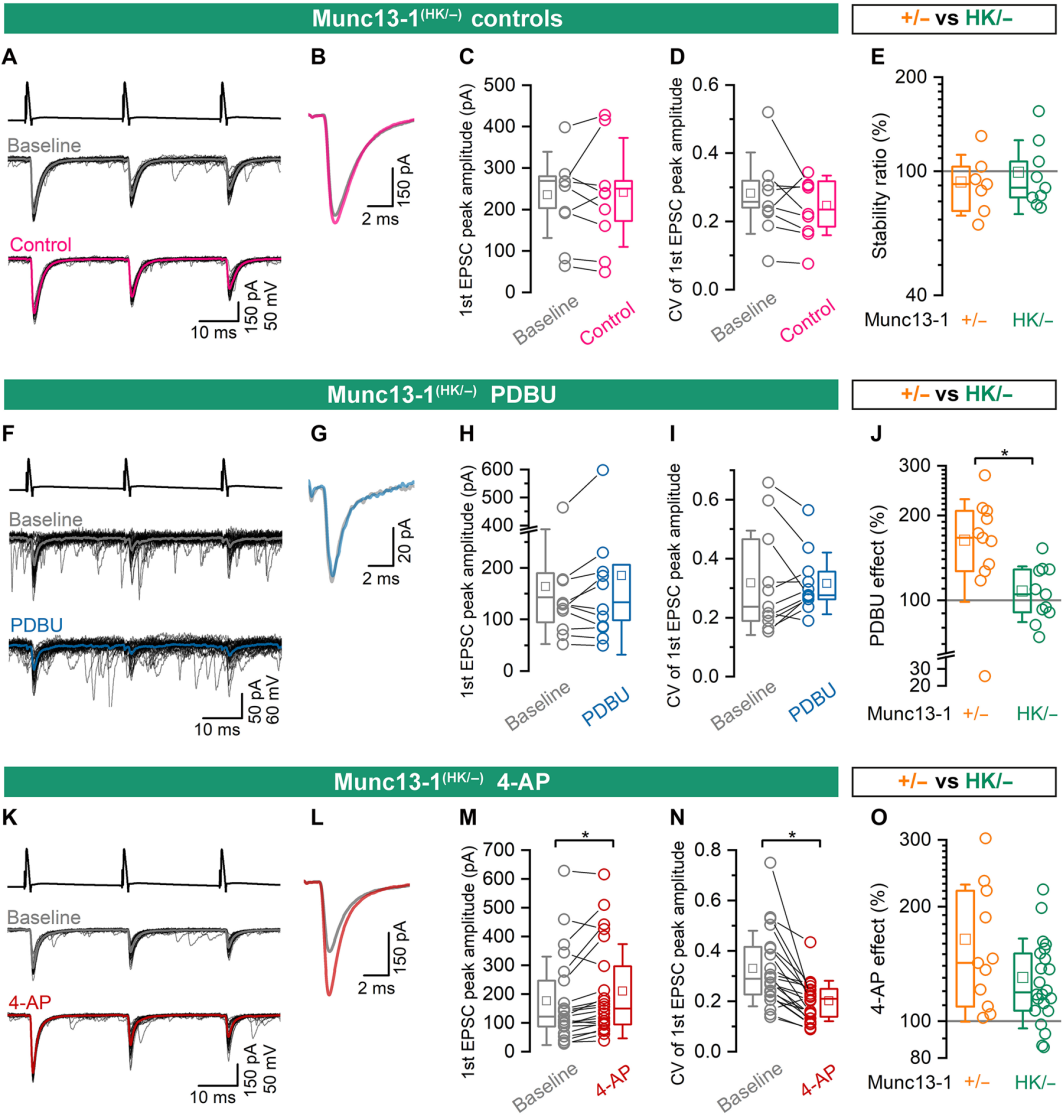
PDBU has no effect on Munc13-1^(HK/−)^ mutated CA1PC-FSIN synapses. (**A**) A stability control experiment performed on a Munc13-1^(HK/−)^ CA1PC-FSIN pair. Three APs at 40 Hz were evoked in the PC (top black trace), generating eEPSCs in the FSIN (individual traces, black; average, gray). The protocol, which involved no drug application, consisted of a 3-min baseline, a 10-min resting interval (“wash in period”), and a final 3-min recording (bottom traces). (**B**) Superimposed averaged first eEPSCs from baseline (gray) and control (pink) for the pair shown in (A). (**C** and **D**) The mean peak amplitudes [*P* = 0.81, Wilcoxon signed rank test (W test) (C)] and the CV [*P* = 0.19, W test (D)] of the first eEPSCs are not significantly different in controls compared to baseline (*n* = 9). (**E**) Box plot showing the stability of the first eEPSCs in Munc13-1^(+/−)^ (orange; *n* = 7) and Munc13-1^(HK/−)^ (green) CA1PC-FSIN connections. There is no significant difference (*P* = 0.75, MW test) between the genotypes. (**F** to **J**) The same as (A) to (E), but after the baseline period, 1 μM PDBU was applied. The amplitude (H) and the CV (I) of the first EPSCs did not change significantly (*P* = 0.31 and 0.89, respectively, W test), but the effect of PDBU (J) is significantly larger in Munc13-1^(+/−)^ compared to Munc13-1^(HK/−)^ CA1PC-FSIN connections (*P* = 0.0086, MW test; *n* = 11 in each). (**K** to **O**) The same as (A) to (E), but after the baseline period, 5 μM 4-AP was applied. The amplitude [*P* = 0.0058, W test, (M)] and the CV [*P* = <0.0001, W test, (N)] of the first EPSCs changed significantly after 4-AP application, but its effect does not differ significantly between Munc13-1^(+/−)^ and Munc13-1^(HK/−)^ CA1PC-FSIN connections [*P* = 0.20, MW test; *n* = 11 and 22, respectively, (O)]. *P < 0.05.

First, the stability of eEPSCs at Munc13-1^(HK/−)^ and Munc13-1^(+/−)^ CA1PC-FSIN connections was assessed by recording a baseline period for 3 min, followed by a 10-min period without any stimulation and then a second 3-min period with presynaptic stimulation (Fig. 5, A and B). For the Munc13-1^(HK/−)^ CA1PC-FSIN connections, the first eEPSC amplitude in the second 3-min period was 99 ± 26% (*n* = 9; Fig. 5, C and E) of that recorded in the first 3 min (baseline) without a significant change in CV (Fig. 5D). Similar stability was observed in Munc13-1^(+/−)^ CA1PC-FSIN connections (92 ± 20% of baseline; *n* = 7; Fig. 5E). When the same protocol was applied, but we washed in 1 μM PDBU, eEPSC amplitudes in Munc13-1^(HK/−)^ synapses showed no significant change (107.9 ± 24.2% of baseline; *n* = 11; Fig. 5, F to J). In contrast, PDBU significantly increased eEPSC amplitudes in Munc13-1^(+/−)^ CA1PC-FSIN connections (163.5 ± 64.7% of baseline; *n* = 11; Fig. 5J).

We also tested the effect of 4-AP on Munc13-1^(HK/−)^ CA1PC-FSIN connections, which increases AP-evoked Ca^2+^ influx into presynaptic axon terminals (*8*) and consequently augments SV fusion. 4-AP significantly increased the first eEPSC amplitudes to 130.3 ± 34.6% of baseline (*n* = 22; Fig. 5, K to O). Although this enhancement tended to be smaller than that observed at Munc13-1^(+/−)^ CA1PC-FSIN synapses (164.2 ± 64.6% of baseline; *n* = 11; Fig. 5O), the difference did not reach statistical significance (MW test, *P* = 0.20).

### Similar number of Munc13-1 nanoclusters in AZs in wild-type and Munc13-1 HK synapses

**Fig. 6.**
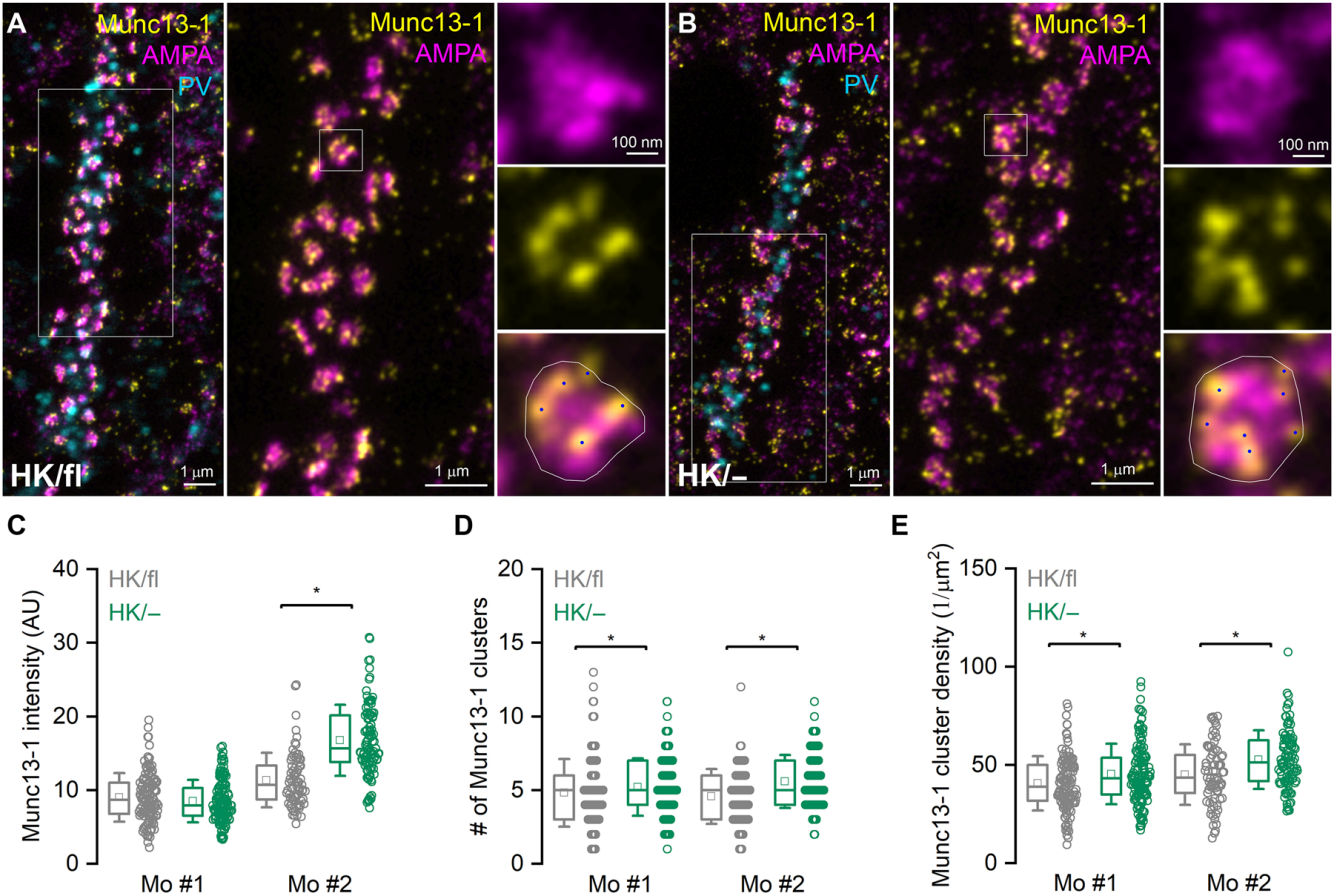
Munc13-1 HK mutation has a modest effect on the number of Munc13-1 nanoclusters in PV^+^ IN dendrite-innervating AZs. (**A** and **B**) Postembedding triple immunofluorescent labeling for PV (cyan, confocal), AMPA receptors (magenta, STED), and Munc13-1 (yellow, STED) in the CA1 stratum oriens of the left (A) and right (B) hemispheres obtained from a Munc13-1^(HK/fl)^ mouse. High concentration of Cre-recombinase expressing AAVs were injected into the right CA1 area. (A) Low (left) and high (middle) magnification images of a PV^+^ dendrite covered with synapses labeled for AMPA receptors and Munc13-1 in the left CA1 where PCs do not express Cre-recombinase [Munc13-1^(HK/fl)^]. An en face view synapse is enlarged in the right. Munc13-1 nanoclusters (blue dots) were identified and counted within the synaptic area (white outline). (B) The same as in (A), but the images were taken in the right CA1 region where PCs express Cre-recombinase [Munc13-1^(HK/−)^]. (**C** to **E**) Mean intensity of Munc13-1 fluorescence (C), number (D), and density (E) of Munc13-1 nanoclusters in en face view synapses on PV^+^ dendrites in Munc13-1^(HK/fl)^ (gray, *n* = 125 in mouse #1, and *n* = 77 in mouse #2) or Munc13-1^(HK/−)^ (green, *n* = 138 in mouse #1, and *n* = 103 in mouse #2). The Munc13-1 intensity was significantly different only in mouse#2 [*P* < 0.0001, MW test, (C)]. The number of Munc13-1 nanoclusters (D) was slightly (15%), but significantly larger in Munc13-1^(HK/−)^ compared to Munc13-1^(HK/fl)^ synapses (mouse #1: HK/fl: 4.8 ± 2.3; HK/−: 5.2 ± 1.9, *P* = 0.03 MW test; mouse #2: HK/fl: 4.6 ± 1.9; HK/−: 5.6 ± 1.8, *P* = 0.0004 MW test). The density of nanoclusters was also significantly higher in Munc13-1^(HK/−)^ compared to Munc13-1^(HK/fl)^ synapses (mouse #1: *P* = 0.018; mouse #2: *P* = 0.002, MW test). AU, arbitrary units. *P < 0.05.

So far, we have provided functional evidence that HK mutation causes an increase in *Pv*, without affecting *N* and *q*. Next, we aimed to provide independent evidence corroborating the lack of change in *N*. It has been convincingly demonstrated that the number of Munc13-1 nanoclusters within presynaptic AZs correlates well with the functionally determined *N* (*49*, *50*). Thus, we turned to postembedding immunofluorescent localization of Munc13-1 in AZs innervating PV-immunopositive (PV^+^) INs and analyzed Munc13-1 immunolabeling using STED microscopy. First, we injected high concentrations of Cre-recombinase encoding AAVs into the dorsal CA1 of two adult Munc13-1^(HK/fl)^/Munc13-2^(−/−)^ mice unilaterally. Three to four weeks postinjection, we transcardially perfused the mice with a paraformaldehyde-containing fixative and sectioned the dorsal hippocampus of both hemispheres into 200-μm-thick coronal slices. We used the noninjected hemisphere as control in both mice. Following dehydration and embedding into Epoxy resin, 500-nm-thick sections were cut and immunolabeled for PV, AMPA receptors, and Munc13-1 (Fig. 6). As expected, PV^+^ dendrites were strongly decorated with AMPA and Munc13-1–immunolabeled synapses in the noninjected, control hemisphere (Fig. 6A). However, despite removing one Munc13-1–coding allele, we observed a qualitatively similar staining pattern in the Cre-injected hemisphere (Fig. 6B). When the reaction was analyzed using STED microscopy, en face view synapses displayed many small nanoclusters for Munc13-1 and a more homogeneous labeling for AMPA receptors (Fig. 6, A and B). The overall intensity of Munc13-1 was similar in one mouse but was significantly enhanced in the other mouse in the injected hemisphere (Fig. 6C). The number of Munc13-1 nanoclusters (for determination, see Materials and Methods) was slightly (~15%), but significantly larger in Munc13-1^(HK/−)^, compared to Munc13-1^(HK/fl)^ synapses (mouse #1: HK/fl: 4.8 ± 2.3, *n* = 125, HK/−: 5.2 ± 1.9, *n* = 138; mouse #2: HK/fl: 4.6 ± 1.9, *n* = 77, HK/−: 5.6 ± 1.8, *n* = 103; Fig. 6D). This slight difference was also observed when we normalized the number of nanoclusters to the area of synapses (Fig. 6E).

Cre recombinase–expressing PCs in these mutant animals express Munc13-1 from a single, HK mutant allele, whereas in the noninjected hemispheres, PCs express Munc13-1 from both alleles. To directly assess a possible gene-dosage effect on Munc13-1 expression, we have repeated these immunolocalization experiments in Munc13-1^(+/fl)^ mice, injecting Cre-expressing AAV into one hemisphere and using the noninjected hemisphere as a control (fig. S2). The intensity of Munc13-1 immunofluorescent reaction was slightly, but significantly lower in Munc13-1^(+/−)^ compared to Munc13-1^(+/fl)^ synapses (fig. S2C). However, this was not accompanied by a change in the number of Munc13-1 nanoclusters (fig. S2D). A small decrease in nanocluster density was also detected (fig. S2E). Our immunolabeling data demonstrate that removing one wild-type Munc13-1 allele does not account for the observed alterations in Munc13-1^(HK/−)^ synapses on PV^+^ dendrites.

### Munc13-1 HK mutation has a more robust effect on CA1PC–O-LM IN than on CA1PC-FSIN connections

**Fig. 7.**
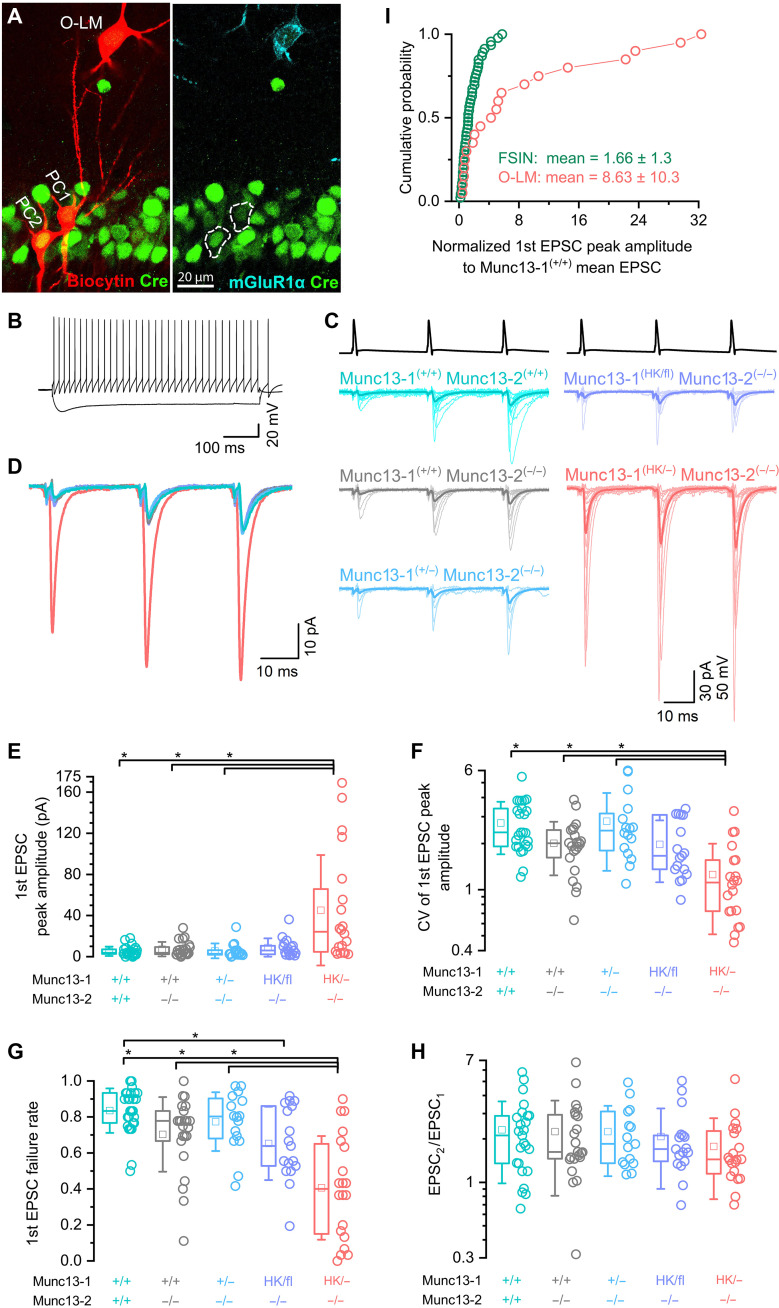
Munc13-1 HK mutation has a dramatic effect on eEPSCs in CA1PC–O-LM INs connections. (**A**) Confocal maximum intensity projection image of two biocytin-filled PCs and an O-LM IN in the hippocampal CA1 region. Both PCs express Cre-recombinase (green), and the IN is immunopositive for metabotropic glutamate receptor 1α (mGLuR1α; right, cyan). Somata of recorded PCs are outlined by dashed lines. (**B**) Membrane potential responses of the O-LM IN shown in (A) to both depolarizing and hyperpolarizing current pulses illustrate its characteristic firing pattern. (**C**) A train of three APs at 40 Hz evoked in a CA1 PC (black traces). Unitary eEPSCs recorded from a synaptically connected O-LM IN are shown below. Color-coded traces represent mice expressing different Munc13-1 and Munc13-2 genotypes. Superimposed averaged eEPSC traces from individual pairs (light traces) and their GTA [dark traces; *n* = 27 (cyan), 23 (gray), 16 (blue), 17 (purple), and 20 (red) recorded pairs] are shown. (**D**) Superimposed GTA traces from (C) highlight differences in eEPSC amplitudes and STP patterns between the different genotypes. (**E** to **H**) First eEPSC mean amplitudes [*P* = 0.0016, 0.03 and 0.002 for red versus blue, gray, and cyan, respectively, (E)], CVs [*P* = 0.00043, 0.045 and < 0.0001 for red versus blue, gray, and cyan, respectively, (F)], failure rates [*P* = 0.0018, 0.028 and < 0.0001 for red versus blue, gray and cyan, respectively, and 0.042 for purple versus cyan, (G)], paired-pulse ratios [no significant difference, (H)] are plotted for the five different Munc13-1 and Munc13-2 genotypes. (**I**) Cumulative probability plots of normalized first eEPSC amplitudes in Munc13-1^(HK/−)^ CA1PC–O-LM and CA1PC-FSIN connections. Evoked EPSC amplitudes for FSIN (green) and O-LM (red) were normalized to the corresponding means of Munc13-1^(+/+)^/Munc13-2^(+/+)^ eEPSC amplitudes. KW test with Dunn’s post hoc test was used in (E) to (H). *P < 0.05.

PC to FSIN synapses are considered as one of the highest *Pv* synapses in cortical and hippocampal networks. The vast majority of hippocampal and cortical synapses have low *Pv* and the postsynaptic responses display short-term facilitation (STF). One of the best-known examples of such low *Pv* synapses is the PC to somatostatin-expressing O-LM IN connection in the hippocampus. In the final set of experiments, we addressed how HK mutation of Munc13-1 affects release at this low-*Pv* synapse by recording CA1PC–O-LM IN connections in Munc13-1^(HK/−)^ animals (Fig. 7). O-LM INs were identified on the basis of their soma shape, location in the stratum oriens and their firing patterns and post hoc immunohistochemical identification of mGluR1α expression (Fig. 7, A and B). Because a previous study from our laboratory demonstrated that Munc13-2 is selectively enriched in presynaptic AZs innervating O-LM INs (*46*), we first assessed the effect of genetic removal of Munc13-2 on the properties of CA1PC–O-LM IN connections. In wild-type mice, the initial eEPSC amplitude in response to a short train of presynaptic APs was small (5.2 ± 4.5 pA, *n* = 27; Fig. 7, C to E, cyan) with high (84%) failure rates (Fig. 7G, cyan). The amplitudes were similarly small in Munc13-1^(+/+)^/Munc13-2^(−/−)^ (7.3 ± 7.0, *n* = 23; Fig. 7, C to E, gray) and in Munc13-1^(HK/fl)^/Munc13-2^(−/−)^ synapses (8.9 ± 8.9, *n* = 17; Fig. 7, C to E, purple). When Cre was injected into the dorsal hippocampus to remove one wild-type allele of Munc13-1 [Munc13-1^(+/−)^/Munc13-2^(−/−)^], eEPSC amplitudes were still similarly small (5.6 ± 7.2, *n* = 16; Fig. 7, C to E, blue). Evoked EPSCs in these conditions showed robust STF (Fig. 7, D and H). However, the amplitudes of the first eEPSCs at Munc13-1^(HK/−)^/Munc13-2^(−/−)^ CA1PC - O-LM connections were significantly larger (45.1 ± 53.7 pA, *n* = 20; Fig. 7, C to E, red) with a significant decrease in CV (Fig. 7F) and failure rate (41 ± 29%, *n* = 20; Fig. 7G). Normalizing the amplitudes of Munc13-1^(HK/−)^/Munc13-2^(−/−)^ CA1PC–OLM IN eEPSCs to the mean of Munc13-1^(+/+)^/Munc13-2^(+/+)^ eEPSC amplitudes revealed an 8.6-fold increase, whereas the same point mutation enhanced the CA1PC-FSIN eEPSCs only by 1.7-fold (Fig. 7I). These results provide an independent line of evidence for differential SV priming as the main mechanism underlying the postsynaptic target cell type–dependent differences in *Pv* in hippocampal networks.

**Fig. 8.**
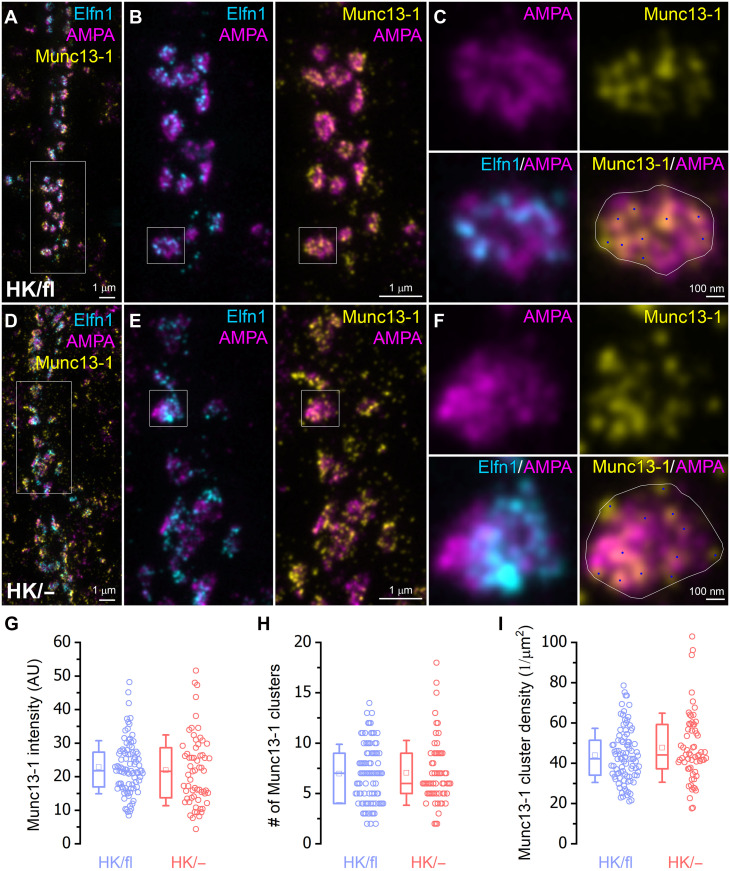
Munc13-1 HK mutation does not affect the number of Munc13-1 nanoclusters at excitatory synapses targeting Elfn1-positive O-LM INs. (**A** to **F**) STED images showing a postembedding triple immunofluorescent labeling for Elfn1 (cyan), AMPA receptors (magenta), and Munc13-1(yellow) in the CA1 stratum oriens of the left [(A) to (C)] and right [(D) to (F)] hemispheres obtained from a Munc13-1^(HK/fl)^ mouse. Injection of Cre-recombinase–containing AAVs into the right CA1 area allowed the selective removal of one wild-type Munc13-1 allele to generate Munc13-1^(HK/−)^ PCs. [(A) to (C)] Low (A) and high (B) magnification images of Elfn1-positive synapses labeled for AMPA receptors and Munc13-1 in the left CA1 region where PCs do not express Cre-recombinase [Munc13-1^(HK/fl)^]. A synapse with en face view is enlarged in (C). Munc13-1 nanoclusters (blue dots) were identified and counted within the synaptic area (white outline). (D and F) The same as in (A) to (C), but the images were taken in the right CA1 region, where PCs express Cre-recombinase [Munc13-1^(HK/−)^]. (**G** to **I**) Mean intensity of Munc13-1 fluorescence [(G) HK/fl: 23 ± 8 AU, HK/−: 22 ± 11 AU, *P* = 0.282], number [(H) HK/fl: 7.0 ± 2.9, HK/−: 7.0 ± 3.2, *P* = 0.872], and density [(I) HK/fl: 43.9 ± 13.5/μm^2^, HK/−: 47.8 ± 17.1/μm^2^, *P* = 0.215] of Munc13-1 nanoclusters in en face Elfn1-positive synapses formed either by Munc13-1^(HK/fl)^ (blue, *n* = 92 synapses) or Munc13-1^(HK/−)^ (red, *n* = 63) PCs. No significant differences were detected in any of the parameters between the two conditions (MW test). AU, arbitrary units.

Last, we analyzed the Munc13-1 content of AZs that innervate O-LM INs (Fig. 8). Here, we used thin sections from one animal used in Fig. 6. Extracellular leucine-reach repeat fibronectin containing 1 (Elfn1) immunoreactivity was used to identify excitatory synapses innervating O-LM INs (*46*, *51*). AMPA receptors and Munc13-1 robustly colocalized with Elfn1 in synapses densely covering dendrites in the stratum oriens and alveus in both noninjected (Fig. 8, A to C) and injected (Fig. 8, D to F) hemispheres. Similar to the AZs innervating PV^+^ INs, en face view of Elfn1-immunopositive synapses contained many small Munc13-1 nanoclusters and a more homogeneous AMPA receptor immunolabeling (Fig. 8, C and F). The overall synaptic intensity of Munc13-1 immunolabeling was similar in control and injected hemispheres (Fig. 8G) as were the numbers of nanoclusters (Fig. 8H) and their densities (Fig. 8I). Despite an 8.6-fold increase in eEPSC amplitude at Munc13-1^(HK/−)^/Munc13-2^(−/−)^ compared to Munc13-1^(+/+)^/Munc13-2^(+/+)^ CA1PC–OLM IN synapses [or ~fivefold increase when comparing Munc13-1^(HK/−)^/Munc13-2^(−/−)^ to Munc13-1^(HK/fl)^/Munc13-2^(−/−)^, which directly matches the genotypes used for immunolabeling in the two hemispheres], there was no significant change in the number of Munc13-1 nanoclusters, consistent with an unaltered *N*. To dissect the confounding effect of removing one wild-type Munc13-1 allele and having the HK mutation on the other one, we repeated these immunolocalization experiments in a Munc13-1^(+/fl)^ mouse in which one hippocampal hemisphere was injected with Cre-containing AAVs (fig. S3). Comparison of the Munc13-1 immunoreactivity between the two hemispheres allows us to test the effect of removing one Munc13-1 allele without having the HK mutation on the other one. The Munc13-1 immunoreactivity was ~20% lower, without any change in the number and density of Munc13-1 nanoclusters (fig. S3, C to E), demonstrating that neither the removal of one allele nor the HK mutation alters the number of Munc13-1 nanoclusters in Elfn1 immunopositive synapses on O-LM INs.

## DISCUSSION

The present work is based on a genetic mouse model, which allows an inducible and selective enhancement of SV priming at central synapses. Specifically, we used mice heterozygous for Munc13-1 HK mutation and for a floxed Munc13-1–coding allele [Munc13-1^(HK/fl)^], which allowed us to eliminate the floxed wild-type Munc13-1 allele in CA1PCs following viral expression of Cre recombinase. Our functional data provide evidence that one Munc13-1–coding wild-type allele [Munc13-1^(+/−)^] is sufficient to ensure normal synaptic function in hippocampal synapses. We also show that removing one Munc13-1 allele did not result in a corresponding ~50% decrease in the overall Munc13-1 level in AZs innervating PV^+^ FSINs or O-LM INs. Instead, synaptic Munc13-1 immunoreactivity was only slightly lower in synapses made by Munc13-1^(+/−)^ PC or remained unchanged or even slightly increased in synapses established by Munc13-1^(HK/−)^ PCs, but the number of Munc13-1 nanoclusters was practically the same. Regulation of Munc13-1 expression levels has recently emerged as a critical determinant of disease states in neurodegenerative and neurodevelopmental disorders (*40*, *52*, *53*). Individuals carrying certain genetic variants of *UNC13A* gene (encoding the Munc13-1 protein), that lead to a reduction of Munc13-1 protein to 20 to 30% of wild-type levels, suffer from a severe neurodevelopmental disorder characterized by a strong reduction in motor performance and epilepsy, while heterozygous individuals are apparently healthy. Moreover, lower Munc13-1 levels facilitate the development of pathology in amyotrophic lateral sclerosis and frontotemporal dementia. We propose that upon the deletion of one Munc13-1 allele, up-regulation of transcription/translation from the remaining allele or a more efficient recruitment of Munc13-1 proteins to the AZ molecular complex occurs. The mechanisms controlling such processes are currently unknown. We should point out that not all synapses on PV^+^ INs in the stratum oriens originate from local collaterals of CA1PCs, but Schaffer collaterals and entorhinal cortical PCs also provide synaptic inputs to these INs (*54*, *55*). Since our anatomical analysis involved random sampling of synapses on these INs, our data are composed of AZs originating from multiple inputs, complicating the interpretation of our results. However, O-LM INs in the CA1 region receive glutamatergic synaptic inputs exclusively from the local collaterals of CA1PCs (*46*, *56*). Therefore, we conclude that the lack of change in Munc13-1 immunolabeling intensity, number, and density of Munc13-1 nanoclusters in Munc13-1^(HK/−)^ PC synapses apply to Cre-expressing CA1PC AZs.

When we tested the function of the HK mutant allele in the presence of a wild-type allele [Munc13-1^(HK/fl)^], we did not detect measurable changes in glutamate release or STP. This result is somewhat unexpected, for several reasons. A study in cultured neurons (*43*) reported a gain of function in synaptic transmission in heterozygous HK/+ neurons. Furthermore, it has been reported that a heterozygous genetic variant in the human *UNC13A* gene that abolished DAG binding (similar, but not identical to the HK mutation) causes a mild form of a neurodevelopmental condition (*40*). The absence of phenotypes we documented here in the Munc13-1^(HK/fl)^ synapses may therefore be associated with the particular synapse subtypes we study, with differences in the contribution of DAG-related signaling in diverse experimental systems, or argues for a minor synaptic change could be amplified to significant alteration at the network level.

Our quantal analysis of postsynaptic responses recorded from wild-type and Munc13-1 HK–mutated CA1PC-FSIN connections revealed that the increase in eEPSC amplitudes is the consequence of a selective increase in *Pv*, without any change in *N* and *q*. It has been suggested that Munc13-1 could affect fusion pore opening during exocytosis and consequently regulate *q* (*57*). Our quantal analysis clearly demonstrates a lack of significant change in *q* in HK-mutated synapses, suggesting that this functional role of Munc13-1 is not modulated by DAG through the C1 domain. The effect of our genetic manipulation on *N* was less predictable. First, our approach of removing one allele could have resulted in an ~50% reduction in the amount of Munc13-1 protein at the AZs, resulting in a dramatic decrease in SV priming and/or *N* (*58*). Alternatively, more Munc13-1 protein could have been recruited to AZs of synapses expressing the Munc13-1 HK mutation, thereby increasing *N* (*59*). We have performed two independent series of experiments to assess potential HK mutation–induced changes in *N*. Paired recordings and subsequent quantal analysis demonstrated a lack of change in *N*, and high-resolution immunolocalization of Munc13-1 in the AZs demonstrated a small, ~15% increase in the number of nanoclusters at CA1PC-FSIN synapses and the lack of change in nanoclusters at CA1PC–O-LM IN synapses. Thus, the 8.6-fold–elevated EPSC amplitude at CA1PC–O-LM IN synapses is paralleled by a total lack of change in the number of Munc-13-1 nanoclusters, which have been shown to positively correlate with *N* (*49*, *50*, *60*). In summary, our results indicate that Munc13-1 HK mutation and, likely, PDBU/DAG-mediated Munc13-1 regulation, enhance postsynaptic responses solely by increasing *Pv*, while leaving *N* and *q* unaffected.

We fitted a sequential, two-step priming model (*7*) to postsynaptic responses evoked by a complex train of presynaptic APs in wild-type and Munc13-1^(HK/−)^ CA1PCs. Our modeling demonstrated that adjusting only the second forward rate constant *k*_2_0_ resulted in a better fit than if only *P*_fusion_ was adjusted. When all model parameters were freely optimized, the best fit caused only a 10% increase in *P*_fusion_ but a 45% increase in the fraction of well-primed SVs (i.e., TS fraction). These results are very similar to those obtained at the calyx of Held using the same HK mutation (*44*). Together, these results demonstrate that the Munc13-1 HK mutation results in a dramatic increase in *P*_occupancy_, without affecting *P*_fusion_, *N*, and *q*.

The mammalian genome contains three closely related paralogs that encode Munc13 proteins (*61*), two of which (encoding Munc13-1 and Munc13-2), are expressed in hippocampal PCs (*45*). Experiments in cultured autaptic Munc13-1/2 double knockout neurons suggest that rescue with Munc13-1 or Munc13-2 encoding virus confer different *Pv*s and STP patterns to synapses. In 90% of axon terminals of cultured PCs, Munc13-1–primed SVs have high *Pv*, and the postsynaptic responses displayed STD, while in the remaining boutons, Munc13-2 was present, which conferred low *Pv* and STF of the EPSCs (*45*). On the basis of these observations, it was proposed that high-*Pv* synapses are equipped with Munc13-1, which enables tight docking of SVs, whereas low-*Pv* synapses that display STF use Munc13-2, and vesicles are loosely docked (*5*). In a previous study, we demonstrated that presynaptic AZs of CA1 PCs that innervate somatostatin- and mGluR1α-expressing O-LM INs contain a high density of Munc13-2 in addition to Munc13-1 (*46*), but Cre-dependent removal of the Munc13-2 encoding gene had no detectable functional consequence. This is consistent with the lack of effects of the genetic deletion of Munc13-2 from mouse photoreceptor ribbon synapses (*62*), hippocampal Schaffer collateral synapses (*63*), and calyx of Held synapses (*64*). Here, we compared eEPSC amplitudes at CA1PC–O-LM IN connections between Munc13-1^(+/+)^/Munc13-2^(+/+)^ and Munc13-1^(+/+)^/Munc13-2^(−/−)^ synapses and found no significant difference. These results using conventional constitutive knock out of Munc13-2 are in line with our previous results using Cre-dependent conditional removal of Munc13-2–encoding gene and demonstrate that either Munc13-2 does not contribute to SV priming, or its function is fully compensated by some other molecules.

Previous studies from our laboratory used various approaches to investigate the mechanisms underlying the postsynaptic target cell type–dependent differences in *Pv* and STP in hippocampal circuits (*8*, *9*, *65*). We reported 20 to 40% larger presynaptic [Ca^2+^] transients in boutons innervating FSINs compared to O-LM INs [similar to those reported for cortical synapses by (*66*)], which was the consequence of a higher presynaptic Ca^2+^ channel density (*8*, *65*). This seems to be responsible for the ~1.7-fold larger *P*_fusion_ at CA1PC-FSIN compared to CA1PC–O-LM IN connections. However, our pharmacological manipulations with PDBU (*8*) together with kinetic modeling (*9*) indicated a much larger, 4- to 6-fold difference in SV priming between these two functionally distinct connections. Here, using a third and independent method, we demonstrate that HK mutation of Munc13-1 causes only a 1.7-fold increase in eEPSC amplitudes at CA1PC-FSIN connections, whereas an >8.5-fold increase at CA1PC–O-LM IN connections was seen. This result provides unequivocal evidence that SV priming, rather than fusion is the main difference between these connections. We found that both the CV of the first eEPSC amplitudes (0.34 versus 1.15) and the PPR (0.52 versus 1.72) was still much lower at Munc13-1^(HK/−)^ CA1PC-FSIN compared to CA1PC–O-LM IN connections (cf. Fig. 1J to Fig. 7F and Fig. 1K to Fig. 7H), which is partially due to the estimated ~twofold difference in *P_fusion_*, [0.6 versus 0.3 in (*9*)] and at least another twofold difference in *P*_occupancy_ (~0.7 and ~0.35). These findings suggest the involvement of additional, yet to be characterized mechanisms that account for lower release from PC axon terminals to O-LM INs, even when Munc13-1 C1 domain modulation is maximized. This could involve differential regulation via the C2A or C2B domains of Munc13-1, or SV release modulation by other proteins. Further in vitro functional experiments with targeted genetic manipulations will be needed to explore the remaining unexplained complexity of this intriguing feature of cortical networks.

## MATERIALS AND METHODS

### Animals

Heterozygous genetically modified mice carrying Munc13-1 point mutation [HK; Munc13-1^(HK/+)^] (*43*) were crossbred with Munc13-1 homozygous conditional knockout mice [Munc13-1^(fl/fl)^; *Unc13a*^tm1.2Sud^] (*44*) and with heterozygous Munc13-2 knockout mice [Munc13-2^(+/−)^] (*45*). The animals with the genotype Munc13-1^(HK/fl)^/Munc13-2^(+/+)^ and Munc13-1^(+/fl)^/Munc13-2^(+/+)^ were injected with Cre recombinase–expressing AAV to remove the floxed Munc13-1 allele and leaving either the point mutated or a single wild-type allele active [Munc13-1^(HK/−)^/Munc13-2^(+/+)^ and Munc13-1^(+/−)^/Munc13-2^(+/+)^, respectively; Fig. 1]. Noninjected littermates with Munc13-1^(HK/fl)^/Munc13-2^(+/+)^ or Munc13-1^(HK/+)^/Munc13-2^(+/+)^ genotypes were used as heterozygous controls. In addition, injected littermates with a Munc13-2^(+/−)^ background [Munc13-1^(HK/fl)^/Munc13-2^(+/−)^ or Munc13-1^(+/fl)^/Munc13-2^(+/−)^] were included in Figs. 2 to 5 and fig. S1, along with animals with homozygous Munc13-2^(+/+)^ genotypes. Because Munc13-2 is present in PC axon terminals innervating mGluR1α-positive INs (*46*), injected Munc13-1^(HK/fl)^/Munc13-2^(−/−)^ and Munc13-1^(+/fl)^/Munc13-2^(–/fl)^ animals were also used to investigate transmission at CA1PC–O-LM INs synapses (Fig. 7). In this context, the latter group is the only one where a conditional knockout of one Munc13-2 allele was used [C57BL/6N- *Unc13b^TM1a(KOMP)Wtsi^*/MbpMmucd; RRID:MMRRC_050292-UCD], while all other groups involved a general knockout of Munc13-2. Noninjected littermates with the genotype Munc13-1^(fl/+)^/Munc13-2^(−/−)^, Munc13-1^(fl/fl)^/Munc13-2^(−/−)^, Munc13-1^(HK/fl)^/Munc13-2^(−/−)^, or Munc13-1^(HK/+)^/Munc13-2^(−/−)^ were included as controls. Sixty-four adult [postnatal day 50 (P50) to P121] male and female Munc13-1/Munc13-2 transgenic mice were used for the electrophysiological recordings. For the wild-type controls [Munc13-1^(+/+)^/Munc13-2^(+/+)^], 35 adult (P50 to P89) male and female Chrna2-Cre-tdTomato transgenic mice were used. In these animals, O-LM INs express tdTomato following the crossbreeding of OE25Gsat/Mmucd, (RRID: MMRRC_036502-UCD, on C57BL/6J background) (*67*) with reporter line Ai9 or Ai14 [Gt(ROSA) 26Sor_CAG/LSL_tdTomato]. The animals were housed in the vivarium of the Institute of Experimental Medicine in a normal 12-hour/12-hour light/dark cycle and had access to water and food ad libitum. All the experiments were carried out in accordance with the Hungarian Act of Animal Care and Experimentation 40/2013 (II.14) and with the ethical guidelines of the Institute of Experimental Medicine Protection of Research Subjects Committee (PE/EA/00874-5/2021).

### Virus injection

Mice were anesthetized with a mixture of ketamine, xylasine, pypolphene (0.625, 6.25, and 1.25 mg/ml, respectively, 10 μl/g body weight). Either the pAAV-Ef1a-mCherry-IRES-Cre (Addgene viral prep 55632-AAV8, 1:3 dilution, RRID:Addgene_55632) or pENN.AAV.CamKII 0.4.Cre.SV40 (Addgene viral prep 105558-AAv9; RRID:Addgene_105558, 1:10 dilution) was injected into the dorsal hippocampus (100 nl, at three sites, coordinates from the bregma in millimeters: antero posterior/dorso ventral/lateral: 2/1.6/1, 2.1/1.5/1.1, and 2.2/1.4/1.2). Three weeks after the injection, in vitro acute slices were prepared from the dorsal hippocampus as described below.

### Slice preparation

A total of 99 mice were stably anesthetized with a ketamine, xylazine, and pypolphene cocktail (0.625, 6.25, and 1.25 mg/ml respectively, 10 μl/g body weight) and then decapitated; the brain was quickly removed and placed into an ice-cold cutting solution containing the following (in millimolars): sucrose, 205.2; KCl, 2.5; NaHCO_3_, 26; CaCl_2_, 0.5; MgCl_2_, 5; NaH_2_PO_4_, 1.25; and glucose, 10, saturated with 95% O_2_ and 5% CO_2_. Coronal slices in 250-μm thickness were then cut from the dorsal part of the hippocampus using a Vibratome (Leica VT1200S) and were incubated in a submerged-type holding chamber in artificial cerebrospinal fluid (ACSF) containing the following (in millimolars): NaCl, 126; KCl, 2.5; NaHCO_3_, 26; CaCl_2_, 2; MgCl_2_, 2; NaH_2_PO_4_, 1.25; and glucose, 10; saturated with 95% O_2_ and 5% CO_2_ (pH 7.2 to 7.4) at 36°C for 30 min; and were then kept at 22° to 24°C.

### Electrophysiological recording

Paired whole-cell patch-clamp recordings were performed at 32° to 33°C in ACSF supplemented with 2 μM AM251 to block presynaptic CB1 receptors and 0.35 mM γ-DGG to prevent AMPA receptors saturation up to 6 hours after slicing. Cells were visualized with infrared differential interference contrast imaging on a Nikon Eclipse FN1 or an Olympus BX51 microscope with a 40× water immersion objective [numerical aperture (NA) = 0.8]. CA1 PCs were identified from their position and morphology. FSINs were identified using their position, morphology, and membrane voltage responses to depolarizing or hyperpolarizing current injections (600 ms, from −300 to 800 pA with 50-pA steps). O-LM INs were identified in the stratum oriens of the CA1 region by the tdTomato expression (in Chrna2-Cre-tdTomato animals) and/or somatic morphology and their membrane voltage responses to depolarizing and hyperpolarizing current injections (600 ms, from −300 to 800 pA with 50-pA steps). Putative O-LM IN identity was confirmed by post hoc mGluR1α immunolabeling (*8*, *46*). Patch pipettes (resistance 4 to 6 MΩ for INs and 6 to 12 MΩ for PCs) were pulled from thick-walled borosilicate glass capillaries with an inner filament. The intracellular solution for the INs contained the following (in millimolars): K-gluconate, 130; KCl, 5; MgCl_2_, 2; EGTA, 0.05; creatine phosphate, 10; Hepes, 10; adenosine triphosphate (ATP), 2; guanosine triphosphate (GTP), 1; and biocytin, 7; (pH 7.3); 290 to 300 mosmol. The intracellular solution for the PCs contained the following (in millimolars): K-gluconate, 97.4; KCl, 43.5; MgCl_2_, 1.7; NaCl, 1.8; EGTA, 0.05; creatine phosphate, 10; Hepes, 10; ATP, 2; GTP,0.4; biocytin, 7 and 10 mM glutamate, (pH 7.25); 290 to 305 mosmol. Paired whole-cell recordings were performed while the PCs were held in the current-clamp mode at −65 mV (with a maximum of ±100-pA dc injection), and postsynaptic INs were held at −65 mV in voltage-clamp mode (with a maximum of ±200-pA holding current) with access resistance bellow 20 MΩ with a dual-channel amplifier (MultiClamp 700B; Axon Instruments). APs were evoked in the PC with 1.2- to 1.5-ms-long depolarizing current pulses (2.2 nA) with 5 to 60 s intertrial intervals depending on the protocol. The baseline of eEPSCs in Fig. 2 was recorded by paired-pulse stimulation with 25-ms interstimuli interval and 5-s intertrial intervals. After washing in 5 μM 4-AP and increasing the extracellular [Ca^2+^], paired-pulse followed by a train of 15 APs at 100-Hz protocol was recorded with 5-s intertrial intervals. For model fitting, a complex protocol consisting of 6 AP preconditioning train at 20 Hz, followed by 15 APs at 100 Hz, and a recovery test train of 6 APs at 100 Hz after 110 ms of recovery was recorded with 60-s intertrial intervals. Pharmacology experiments in Fig. 5 were done by recording a baseline (three APs at 40 Hz, 36 traces) for 3 min, and then a 10-min period was allowed for drug wash-in and then recording was continued with the same protocol. Stability controls were recorded without washing in any drugs during the 10 min “wash-in” period. Data were filtered at 3 to 4 kHz (Bessel filter), digitized on-line at 50 kHz, recorded, and analyzed using Clampfit 10.7 and Clampfit 11.4 (Molecular Devices). INs with increased access resistance (>25%) during the recording were excluded.

### Tissue processing after paired whole-cell patch-clamp recording

After recordings, the slices were fixed in a solution containing 4% formaldehyde (Molar Chemicals, Budapest, Hungary), 0.2% picric acid in 0.1 M phosphate buffer (PB) (pH 7.4), at 4°C for 12 hours. Immunolabeling was carried out without resectioning the slices. As described in Holderith *et al.* (*46*), slices were washed in 0.1 M PB and blocked in normal goat serum (NGS, 10%; Vector Laboratories, CA) with 0.3% Triton X-100 for 1 hour made up in tris-buffered saline (TBS, pH 7.4). Slices were then incubated in the solutions of the primary antibodies (Abs): guinea pig anti-mGluR1α (1:1000, Frontier Institute Co. Ltd.; catalog no. mGluR1α-GP-Af660, AB_2531897) or a cocktail of this Ab and a mouse anti-Cre Ab [immunoglobulin G1 (IgG1), 1:1000, Millipore, catalog no. MAB3120, AB_2085748], diluted in TBS containing 2% NGS and 0.3% Triton X-100. Biocytin was visualized with Cy3-conjugated streptavidin (1:1000; Jackson Immunoresearch Laboratories, PA, AB_2337244). After several washes, the following secondary Abs were applied: Alexa Fluor 488–conjugated goat anti-mouse IgG1 (Jackson Immunoresearch, Code: 115-547-185, AB_2632534) and Cy5-conjugated donkey anti-guinea pig IgGs (Jackson Immunoresearch, Code 706-175-148, AB_2340462). Sections were mounted in Vectashield. Image stacks were acquired with an Olympus FV3000 confocal microscope with 20× or 60× (oil immersion) objectives. A PC was considered virally infected if its nucleus had detectable Cre immunosignal. An IN was considered an O-LM cell if its axon arborized in the stratum lacunosum-moleculare and/or it was immunopositive for mGluR1α.

### Quantal analysis

To measure the quantal size, uncontaminated EPSCs (without obvious asynchronous EPSCs) at the end of the 100-Hz train (8th to 17th APs) were extracted, including failures. The integrated charge was measured for these traces (area under these traces) within a 3- to 4-ms window. The integrated charge histogram was binned using a number of bins equals to the square root of the total number of traces extracted. The first bin usually formed the cutoff value separating successful release events from failures. The traces of the first bin were visually inspected. Next, the successful EPSCs were further processed to select presumed uniquantal eEPSCs. The probability of uniquantal release events (*P_1_*) at the end of the train was calculated using the binomial distribution formula

$$P_{1}=N_{\text{init}}\times \mathrm{Pv}\;{\left(1-\mathrm{Pv}\right)}^{N_{\text{init}}-1}$$ (1)

where *N*_init._ is the initial estimate of number of release sites (Eq. 3), *Pv* is the release probability at the end of the train, calculated by dividing the mean amplitude of EPSCs at the end of the train by the largest measured eEPSC at the first AP under high-*Pv* condition. We estimated *N*_init._ as in Eq. 3 from solving Eq. 2 for *N*_init_

$$\mathrm{Pf}={\left(1-\mathrm{Pv}\right)}^{N_{\text{init}}}$$ (2)

$$N_{\text{init}}=\text{ln}\mathrm{Pf}/\text{ln}\left(1-\mathrm{Pv}\right)$$ (3)

where *P*_f_ represents the probability of failures at the end of the train.

The traces were ranked according to the charge values, and the traces correspond to the percentile between (*P*_f_)^th^ and (*P*_f_ + *P_1_*)^th^ was considered as the subgroup of traces where 1 quanta was released. To calculate *q*, this subgroup of eEPSCs were inspected, and the mean was calculated and considered as the *q* (Fig. 2).

The number of release sites (*N*) was calculated by dividing the maximum eEPSC peak amplitude (recorded at first AP under high-*Pv* condition) by the *q* as calculated above. The maximum eEPSC at the first AP in the long train in 5 μM 4-AP and high [Ca^2+^]_e_ was considered as the largest possible eEPSCs because under such high-*Pv* condition, there is a relatively high probability that release will happen at all *N* sites (see Fig. 3, E and F for the probability that SVs are released at all *N*s). Baseline *Pv* and *Pv* under high-*Pv* conditions were determined by dividing the mean amplitude of the first eEPSC measured at baseline and during high-*Pv* period, respectively, by the maximum eEPSC amplitude recorded during the high-*Pv* condition.

MPFA was carried out according to (*47*). The mean and variance of eEPSC peak amplitudes were calculated for the baseline paired-pulse recording (Fig. 2, A and F) and for the first four eEPSCs under high-*Pv* conditions (Fig. 2, B and G). All the extracted traces (as described above) at the end of the 100-Hz train were pooled together and the mean and variance of those were also plotted and used for the parabolic fit (Fig. 3, A and D). Plots of mean versus variance values were fitted with a parabola to determine *N* and *q*. *Pv* was calculated by dividing first eEPSCs mean amplitude by *N* and *q*.

In Fig. 3 (B, C, E, and F), the theoretical binomial distribution probability was calculated using the following formula

Px=(Nx)Pvx(1−Pv)N−x (4)

where *P_x_* is the probability of *x* number of successful release among total number of release sites (*N*) with uniform *Pv* among release sites.

Bootstrapping was used to determine the 95% confidence interval of the binomial distribution at each individual experiment with the exact number of traces recorded (~80 per experiment). Basically, ~80 random resampling has been done from a data pool that has a binomial distribution of certain *Pv* and *N*. This has been repeated 5000 times, and the 0.975 and 0.025 percentiles were calculated and plotted for each *P_x_*. The stability of the recordings was checked for each connection and assessed by Spearman correlation, and only stable recordings (*P* > 0.05) were used to determine quantal parameters.

### Modeling STP

A sequential, two-step priming model (*7*) was implemented in Berkeley Madonna as in (*9*). Michaelis-Menten–like saturation for *k*_1_ in response to [Ca^2+^] was implemented, but the Ca^2+^ dependence of *P*_fusion_, as described in Lin *et al.* (*7*), was omitted. In addition to LS and TS, a “Labile Tight State” (TSL), which contributes to release at high frequencies, was also implemented. The priming rate constants *k*_1_ and *k*_2_ are Ca^2+^ dependent, with resting values *k*_1_0_ and *k*_2_0_, and linear slope factors σ*_1_* and σ*_2_* defining their Ca^2+^ sensitivity. The unpriming rate constants *b*_1_ and *b*_2_ are set to fixed values. The two parameters describing TSL were κ, the fraction of LS SVs transferred to TSL per AP and a backward rate constant of *b*_3_. The resting [Ca^2+^] was constrained to 50 nM, and the increment of effective [Ca^2+^] following each AP was constrained to 110 nM as in (*7*), and they were kept constant during fitting. To quantify the “goodness of fit,” the RMSD was calculated for each fit. Euler’s integration method was used as the numerical procedure for solving the differential equations. The model parameters were optimized to fit the experimental data using Nelder-Mead simplex method, which Berkeley Madonna software uses (*68*), and it aims to find parameter values that minimize the RMSD between the experimental data points and the model solution over the specified time interval. EPSC amplitudes were converted to quantal content, by dividing the peak amplitudes by the estimated quantal size [see (*9*)].

### RRP calculation

The quantal content of the high-*Pv* train (data from Fig. 2, B and G) was calculated for each CA1PC-FSIN connection by dividing the eEPSC peak amplitude by its corresponding calculated *q*. Cumulative quanta content plots were then generated for each CA1PC-FSIN connection. The SMN method (*48*) was used to estimate the RRP size by extrapolating the linear fit of the cumulative release at the last six stimuli. The *y* axis intercept of this linear fit was defined as the RRP size.

### Tissue processing and postembedding immunofluorescent labeling

Two (P77 and a P82) male Munc13-1^(HK/fl)^/Munc13-2^(–/–)^ mice were injected with the pAAV-Ef1a-mCherry-IRES-Cre virus only in the right dorsal hippocampus at P56. One (P110) female Munc13-1^(+/fl)^/Munc13-2^(−/−)^ mouse was injected with the pAAV-CAMKII-Cre virus only in the right dorsal hippocampus at P86. The mice were deeply anesthetized and were transcardially perfused with ice-cold fixative containing 4% formaldehyde (Molar Chemicals, Budapest, Hungary), 0.2% picric acid in 0.1 M PB (pH 7.3) for 10 min. Coronal forebrain sections (200 μm thick) were cut from the dorsal hippocampus with a Vibratome. Sections from the Munc13-1^(+/fl)^/Munc13-2^(−/−)^ mouse were incubated with a mouse anti-Cre Ab (IgG1, 1:500, Millipore, catalog no. MAB3120, AB_2085748) diluted in TBS containing 2% NGS and 0.3% Triton X-100. Cre labeling was visualized with an Alexa Fluor 488–conjugated goat anti-mouse IgG1 Ab (1:500, Jackson Immunoresearch, Code: 115-547-185, AB_2632534). Epifluorescent images were taken from the hippocampal areas of the virus-injected right and the noninjected left hemispheres to confirm Cre expression (mCherry or Alexa Fluor 488) in the right and its absence in the left hemisphere. Right and left hippocampal sections from the same rostrocaudal positions were selected and processed in parallel. Blocks were dehydrated in a graded series of ethanol, incubated in acetonitrile, and finally infiltrated with the epoxy resin (Durcupan). Five hundred–nanometer–thick sections were cut using a Histo Jumbo diamond knife (Diatome) and mounted onto gelatin-coated coverslips. Etching the resin, antigen retrieval, immunolabeling, and elution were carried out as reported previously (*69*). The resin was etched with Na-ethanolate for 6 min and rinsed in 96% ethanol three times and then with distilled water. Retrieval was carried out in 0.02 M tris base (pH 9) containing 0.5% SDS for 60 min at 80°C. After several washes in TBS containing 0.05 to 0.1% Triton X-100 (TBST, pH 7.6), sections were blocked in TBST containing 6% BlottoA (Santa Cruz Biotechnology), 10% NGS (Vector Laboratories), and 1% bovine serum albumin (Sigma-Aldrich) for 30 min and then incubated in primary Abs diluted in blocking solution at room temperature overnight. Multiplexed postembedding reactions were carried out in two sequential labeling rounds. Mixture of the following Abs were used in the first labeling round: either a rabbit anti-PV (1:200, Thermo Fisher Scientific, #PA1-933, RRID: AB_2173898) or a rabbit anti-Elfn1 (1:200, Synaptic Systems, catalog no. 448 005, RRID:AB_3662607) Ab mixed with a guinea pig anti–pan-AMPAR (1:200, Frontier Institute, catalog no. panAMPAR-GP, RRID: AB_2571610) and a mouse anti-Kv2.1 (1:200, Neuromab, catalog no.75-014, RRID: AB_10673392) Ab. After several washes, the sections were incubated in the following secondary Abs: donkey anti-rabbit IgG–coupled Alexa Fluor 488 [1:200, Jackson ImmunoResearch Labs, catalog no. 711-545-152, RRID:AB_2313584), goat anti-rabbit IgG–coupled Abberior Star 635P (1:200, Abberior, catalog no. ST635P-1002-500UG, RRID:AB_2893229)], goat anti-guinea pig IgG–coupled with Abberior STAR 580 (1:100, Abberior, catalog no. ST580-1006-500UG), a subclass 1 specific, goat anti-mouse IgG–coupled with Alexa Fluor 647 (1:200, Jackson ImmunoResearch Labs, catalog no. 115-605-205, RRID:AB_2338916), or Alexa Fluor 488 (Jackson ImmunoResearch Labs, catalog no. 115-545-205, RRID:AB_2338854). After labeling, the sections were washed and mounted in Slowfade Diamond Antifade (Thermo Fisher Scientific, Waltham, MA). Low-magnification confocal images were taken using a 20× objective (FV3000, Olympus Europe) and matched with the images taken of the same block showing the Cre-recombinase expression to create a map. High-magnification confocal and STED images were then taken in the stratum oriens of the injected right and noninjected left CA1 areas with an Abberior Instruments Facility Line STED microscope (60 × 1.42 NA objective, pixel size = 20 nm). For sequential labeling, the STED imaging step was followed by an Ab elution step. The immunolabeling was removed through a 7-min incubation in TBST containing 1% SDS (pH 7.7) at 80°C. This elution step has been shown to efficiently abolish >98% of the immunolabeling (*69*). After a 5-min wash in TBST, a second round of immunolabeling was performed. The rabbit Munc13-1 (1:200, Synaptic Systems, catalog no. 126 103, RRID: AB_887733) Ab was mixed with Abs against Kv2.1 and AMPA receptors to identify the same region and synapses captured after the first labeling round. Kv2.1, AMPA receptors, and Munc13-1 were visualized by secondary Abs conjugated to Alexa Fluor 488, Abberior STAR 580, and Abberior STAR 635P, respectively. Sections were again mounted in Slowfade Diamond on gelatin-coated coverslips. Confocal and STED images were then acquired. Images taken of the same areas in both rounds were aligned in Fiji software based on the AMPA receptor labeling. Images were taken with the same acquisition settings through the two rounds.

### Analysis of the Munc13-1 nanoclusters

Aligned PV/Elfn1, AMPA, and Munc13-1 images were analyzed in Fiji software using custom written macros. Synapses with round, spatially expanded AMPA receptor fluorescence and overlapping Munc13-1 signal were classified as en face synapses and were included in the analysis. Areas containing PV^+^ or Elfn1^+^ en face synapses were manually outlined for further segmentation. First, within the outlined areas, a binary synaptic mask was created for excitatory synapses based on the fluorescent signal for AMPA receptors. A Gaussian filter (radius = three pixels) was applied to the AMPA receptor channel, followed by an automatic Otsu thresholding to create a mask for the AMPA receptor signal. To minimize the joint detection of nearby synapses, a second segmentation mask was also created using the find maxima function (FM, output: segmented particles). Combination of the two binary masks resulted in the final synaptic mask. For the detection of Munc13-1 nanoclusters, synaptic masks were slightly dilated (by four pixels) to include complete presynaptic AZs. The Munc13-1 fluorescent signal accumulated in high-intensity puncta within synapses, consistent with the formation of Munc13-1 nanoclusters in the AZs. Munc13-1 intensity peaks were detected and counted within synaptic masks using the FM function (output: point selection). Measurements were performed in background-subtracted images. The background was determined by the rolling ball algorithm (radius = seven pixels).

### Statistical analysis

Data are presented as means ± SD throughout the manuscript, and “*n*” represents number of recorded cell pairs or synapses as appropriate unless stated otherwise in the text. Shapiro-Wilk test was used to test the normality of our data. The following statistical tests were used unless otherwise stated: to compare two dependent groups, Wilcoxon signed-rank test was used; to compare two independent groups, MW test was used. To compare multiple populations of data, Kruskal-Wallis test was used followed by Dunn’s test. Statistical tests were performed in OriginPro (2020b, OriginLab) statistical significance was assessed at *P* < 0.05.
