# Supplementary material for: Selective, genetically induced increase in synaptic vesicle priming
Source: Sci Adv. 2026 Apr 8;12(15):eaee6848. doi: 10.1126/sciadv.aee6848 (PMC13060592; doi:10.1126/sciadv.aee6848)
Supplement: Supplementary file 1 — Figs. S1 to S3 Table S1 [file sciadv.aee6848_sm.pdf]

Science Advances
AAAS

Supplementary Materials for

Selective, genetically induced increase in synaptic vesicle priming

Mohammad Aldahabi *et al.*

Corresponding author: Zoltan Nusser, nusser@koki.hu

Sci. Adv. **12**, eaee6848 (2026)
DOI: 10.1126/sciadv.aee6848

This PDF file includes:

Figs. S1 to S3
Table S1

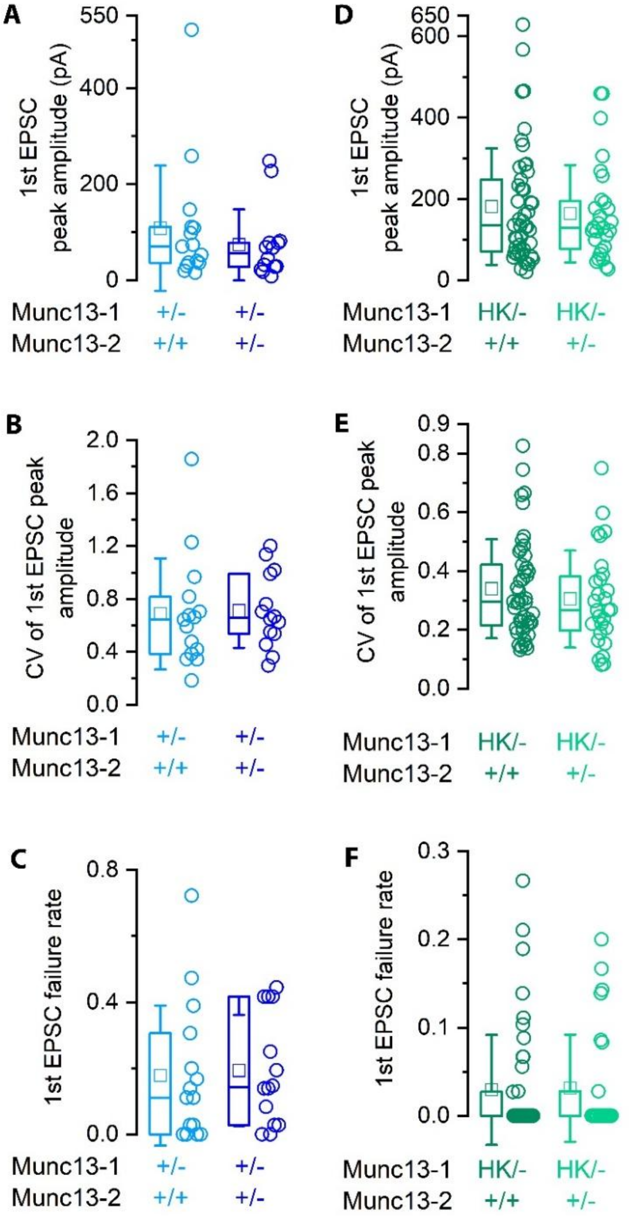

Figure S1. Evoked EPSCs at PC – FSINs do not differ between the Munc13-2$^{(+/-)}$ and Munc13-2$^{(+/+)}$ backgrounds.

(A) Mean peak amplitudes of the 1st eEPSC are not significantly different in Munc13-1$^{(+/-)}$/Munc13-2$^{(+/+)}$ (light blue, n = 15) compared to Munc13-1$^{(+/-)}$/Munc13-2$^{(+/-)}$ (dark blue, n = 14, p = 0.35).

(B) Coefficient of variation values of the first eEPSC amplitudes are not significantly different in in Munc13-1$^{(+/-)}$/Munc13-2$^{(+/+)}$ (light blue, n = 15) compared to Munc13-1$^{(+/-)}$/Munc13-2$^{(+/-)}$ (dark blue, n = 14, p = 0.59).

(C) Failure rates are not significantly different in Munc13-1$^{(+/-)}$/Munc13-2$^{(+/+)}$ (light blue, n = 15) compared to Munc13-1$^{(+/-)}$/Munc13-2$^{(+/-)}$ (dark blue, n = 14, p = 0.52).

(D - F) Same as (A – B) but Munc13-1$^{(HK/-)}$/Munc13-2$^{(+/+)}$ (dark green, n = 46) is compared to Munc13-1$^{(HK/-)}$/Munc13-2$^{(+/-)}$ (light green, n = 28) with p values = 0.72 (D), 0.45 (E) and 0.95 (F), respectively.
MW-test was used in all statistical comparisons.

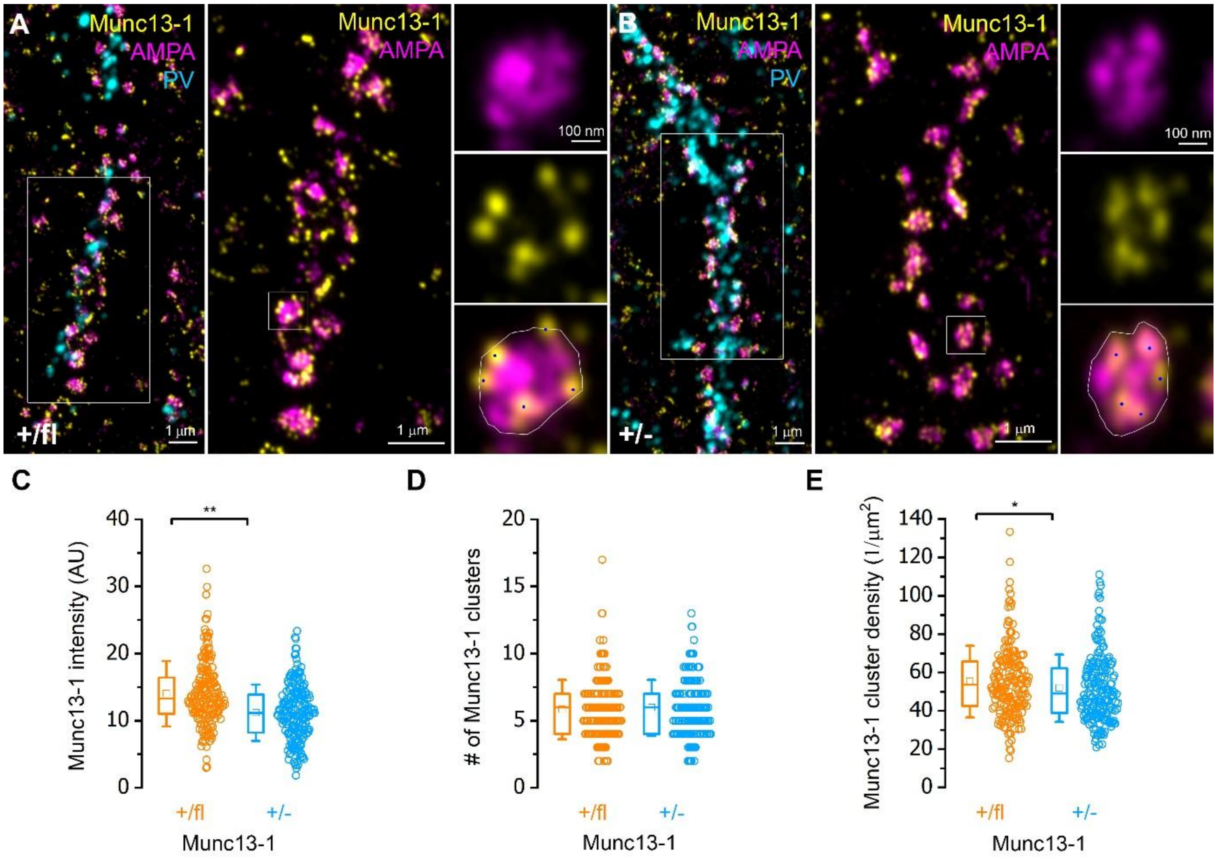

Figure S2. Single allele expression of Munc13-1 has no effect on the number of Munc13-1 nanoclusters at excitatory synapses on PV+ IN dendrites

(A-B) Postembedding triple immunofluorescent labeling for PV (cyan, confocal), AMPA receptors (magenta, STED) and Munc13-1 (yellow, STED) in the CA1 stratum oriens of the left (A) and right (B) hemispheres obtained from a Munc13-1$^{+/fl}$ mouse. High concentration of Cre-recombinase expressing AAVs were injected into the right CA1 area. Spatially restricted virus injection ensured the Munc13-1 expression from a single allele (Munc13-1$^{(+/-)}$) only in the PCs of the right CA1 area.

(A) Low (left) and high (middle) magnification images of a PV+ dendrite covered with synapses labeled for AMPA receptors and Munc13-1 in the left CA1 where PCs do not express Cre-recombinase (Munc13-1$^{(+/fl)}$). An *en face* view synapse is enlarged in the right. Munc13-1 clusters (blue dots) were identified and counted within the synaptic area (white outline).

(B) Same as in (A), but the images were taken in the right CA1 region where PCs express Cre-recombinase (Munc13-1$^{(+/-)}$).

(C-E) Mean intensity of Munc13-1 fluorescence (C), number (D) and density (E) of Munc13-1 nanoclusters in *en face* view synapses on PV+ dendrites in Munc13-1$^{(+/fl)}$ (orange, n = 187) or Munc13-1$^{(+/-)}$ conditions (blue, n = 196). The synaptic Munc13-1 intensity was slightly, but significantly lower in Munc13-1$^{(+/-)}$ (Munc13-1$^{(+/fl)}$: 14 ± 4.9 AU; Munc13-1$^{(+/-)}$: 11.2 ± 4.2 AU, p = 2.6E-8, MW-test). No significant difference was detected in the number of Munc13-1 nanoclusters (D) between the two conditions (Munc13-1$^{(+/fl)}$: 5.8 ± 2.2; Munc13-1$^{(+/-)}$: 6.0 ± 2.1, p = 0.429 MW-

test). The density of Munc13-1 nanoclusters was slightly smaller in the Munc13-1$^{(+/-)}$ synapses (Munc13-1$^{(+/fl)}$: 55.3 ± 18.6, Munc13-1$^{(+/-)}$: 51.8 ± 17.5, p = 0.034, MW-test).

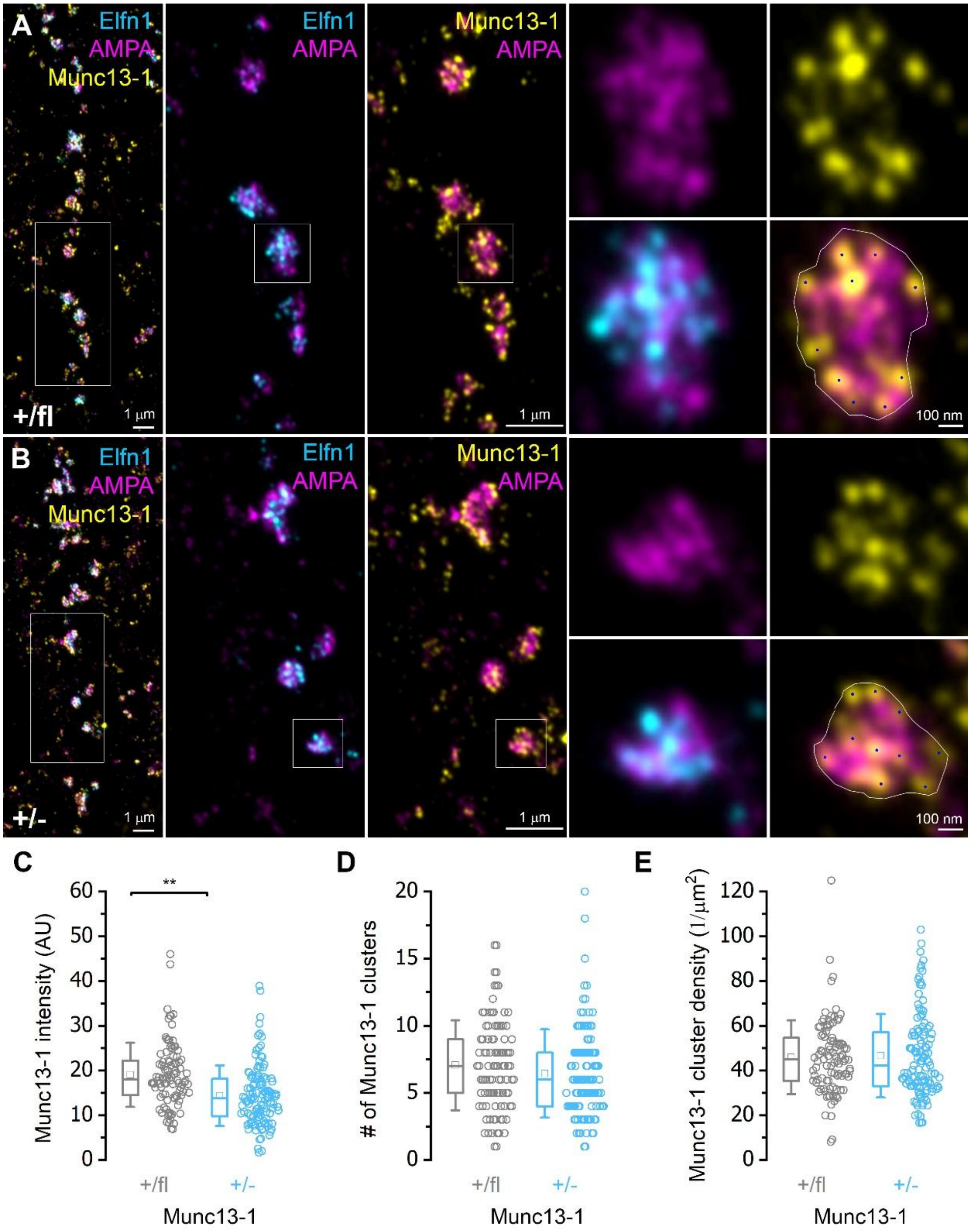

Figure S3. Single allele expression of Munc13-1 has no effect on the number of Munc13-1 nanoclusters at excitatory synapses targeting Elfn1 positive O-LM INs

(A-B) Fluorescent images showing a postembedding triple immunofluorescent labeling for Elfn1 (cyan), AMPA receptors (magenta) and Munc13-1(yellow) in the CA1 stratum oriens of the left (A) and right (B) hemispheres obtained from a Munc13-1$^{+/fl}$ mouse. High concentration of Cre-recombinase expressing AAVs were injected into the right CA1 area. Spatially restricted virus

injection ensured the Munc13-1 expression from a single allele (Munc13-1$^{(+/-)}$) only in the PCs of the right CA1 area.

(A) Low (left) and high (middle) magnification images of Elfn1 positive synapses labeled for AMPA receptors and Munc13-1 in the left CA1 region where PCs do not express Cre-recombinase (Munc13-1$^{(+/fl)}$). A synapse with *en face* view is enlarged on the right. Munc13-1 nanoclusters (blue dots) were identified and counted within the synaptic area (white outline).

(B) Same as in (A), but the images were taken in the right CA1 region, where PCs express Cre-recombinase (Munc13-1$^{(+/-)}$).

(C-E) Mean intensity of Munc13-1 fluorescence (C, Munc13-1$^{(+/fl)}$): 19 ± 7.2 AU, Munc13-1$^{(+/-)}$): 14.4 ± 6.8 AU, p = 3.1E-7, MW-test), number (D, Munc13-1$^{(+/fl)}$): 7.1 ± 3.4, Munc13-1$^{(+/-)}$): 6.4 ± 3.3, p = 0.111, MW-test) and density (E, Munc13-1$^{(+/fl)}$): 46 ± 16.5 1/μm^2, Munc13-1$^{(+/-)}$): 46.6 ± 18.7 1/μm^2, p = 0.687, MW-test) of Munc13-1 nanoclusters in *en face* Elfn1 positive synapses formed either by Munc13-1$^{(+/fl)}$ (gray, n = 97 synapses) or Munc13-1$^{(+/-)}$ (light blue, n = 118) PCs.

Parameter abbreviation	Parameter full name	Munc13-1$^{(+/+)}$ (Aldahabi et al., 2024)	Mun13.1 $^{(HK/-)}$	Ratio (Munc13.1$^{(HK/-)}$ / Munc13.1$^{(+/+)}$)
P-fusion	Initial fusion probability	0.60	0.66	1.10
k_{1_0}	Rate constant for the ES → LS transition at rest (ms^{-1})	6.1E-04	5.01E-04	0.82
b_1	Rate constant for the ES ← LS transition (ms^{-1})	3.8E-04	5.32E-04	1.40
k_{2_0}	Rate constant for the LS → TS transition at rest (ms^{-1})	2.4E-04	7.08E-04	2.95
b_2	Rate constant for the LS ← TS transition (ms^{-1})	3.0E-04	3.97E-04	1.32
TSLfrac (κ)	Fraction of LS transferred to TSL by an AP	0.18	0.27	1.46
TauTSL ($1/b_3$)	Decay time constant back from the TSL state (ms)	72.5	39.2	0.54
s_1	Fraction of empty sites transferred to LS subpool by an AP	0.12	0.04	0.36
s_2	Fraction of LS subpool transferred to TS subpool by an AP	0.25	0.32	1.29
$K_{0.5}$	Michaelis-Menten K_D of 1st priming step (μM)	0.33	0.34	1.05
N_{tot}	Total number of release sites	25.5	25.5	
$\Delta[Ca^{2+}]$	$[Ca^{2+}]$ increment per AP-induced 'effective' $[Ca^{2+}]$ transient (μM)	0.11	0.11	
$[Ca^{2+}]$ decay	Decay time constant of the AP-induced 'effective' $[Ca^{2+}]$ transient (ms)	102.5	102.5	
$[Ca^{2+}]$ rest	Basal $[Ca^{2+}]$ at rest (μM)	0.05	0.05	
**RMSD**		**0.00244**	**0.00182**	
$SP_{TS,rest}$	Size of fusion-competent or tightly-docked SV subpool at rest	8.42	11.82	1.40
$SP_{LS,rest}$	Size of fusion-incompetent or loosly-docked SV subpool at rest	10.52	6.63	0.63
$N_{ES,rest}$	Number of empty release sites at rest	6.56	7.05	1.07
TS fraction	Fraction of SVs in the TS state out of the docked SVs (LS+TS)	**0.44**	**0.64**	1.44

Table S1. Parameters of the sequential, two-step priming model and the ratios between the parameters in HK mutant and wild-type controls.
